# Conducting Polymers for Optoelectronic Devices and Organic Solar Cells: A Review

**DOI:** 10.3390/polym12112627

**Published:** 2020-11-09

**Authors:** Ary R. Murad, Ahmed Iraqi, Shujahadeen B. Aziz, Sozan N. Abdullah, Mohamad A. Brza

**Affiliations:** 1 Department of Chemistry, University of Sheffield, Sheffield S3 7HF, UK; ary.murad@charmouniversity.org; 2 Department of Pharmaceutical Chemistry, College of Medical and Applied Sciences, Charmo University, Chamchamal, Sulaimani 46023, Iraq; 3 Hameed Majid Advanced Polymeric Materials Research Lab., Department of Physics, College of Science, University of Sulaimani, Qlyasan Street, Sulaimani 46001, Iraq; 4 Department of Civil engineering, College of Engineering, Komar University of Science and Technology, Sulaimani 46001, Iraq; 5 Department of Chemistry, College of Science, University of Sulaimani, Qlyasan Street, Kurdistan Regional Government, Sulaimani 46001, Iraq; sozan.abdulla@univsul.edu.iq; 6 Department of Manufacturing and Materials Engineering, Faculty of Engineering, International Islamic University of Malaysia, Kuala Lumpur, Gombak 53100, Malaysia; mohamad.brza@gmail.com

**Keywords:** conjugated polymers, oxidative polymerizations, condensation polymerization, Yamamoto coupling reactions, organic photovoltaics, HOMO-LUMO energy levels, architecture of polymer solar cells

## Abstract

In this review paper, we present a comprehensive summary of the different organic solar cell (OSC) families. Pure and doped conjugated polymers are described. The band structure, electronic properties, and charge separation process in conjugated polymers are briefly described. Various techniques for the preparation of conjugated polymers are presented in detail. The applications of conductive polymers for organic light emitting diodes (OLEDs), organic field effect transistors (OFETs), and organic photovoltaics (OPVs) are explained thoroughly. The architecture of organic polymer solar cells including single layer, bilayer planar heterojunction, and bulk heterojunction (BHJ) are described. Moreover, designing conjugated polymers for photovoltaic applications and optimizations of highest occupied molecular orbital (HOMO)–lowest unoccupied molecular orbital (LUMO) energy levels are discussed. Principles of bulk heterojunction polymer solar cells are addressed. Finally, strategies for band gap tuning and characteristics of solar cell are presented. In this article, several processing parameters such as the choice of solvent(s) for spin casting film, thermal and solvent annealing, solvent additive, and blend composition that affect the nano-morphology of the photoactive layer are reviewed.

## 1. Introduction

In the past few decades, a large increase in energy demand to use fossil energy resources, regarding their storage deficiency and environmental impact, has become a worldwide energy issue [1]. Collecting energy from daylight by photovoltaic technologies is considered to be one of the best imperative techniques to overcome increasing global energy demand [2]. Amongst numerous available technologies, photovoltaics (PVs) are considered to be the cleanest way to achieve the desirable goals from the solar energy conversion point of view [3]. Conjugated polymers and polar polymers are used to produce numerous PV devices. For the most part, polar polymers have been used as host electrolytes for applications in electrochemical and dye-sensitized solar cell (DSSC) devices [1,2,4,5,6,7,8,9,10,11,12]. Moreover, conjugated polymers are broadly employed in fabricating organic solar cells (OSCs). Third generation solar cells have been fabricated to pursue low costs and high power conversion efficiencies (PCE) for different applications, for example organic PVs (OPVs), light condensed cells, dye-sensitized solar cells (DSSCs), tandem cells, and organic–inorganic hybrid (OIH) solar cells. Generally, the lifetime and PCE of solar cells comprising organic components have not competed with those of conventional first and second generation solar cells. However, OSCs have been attracting significant attention due to their flexibility, light weight, and low construction cost, which make them appropriate for numerous applications [13].

In recent decades, the development of conjugated polymers and organic semiconductors has led to potential applications in optoelectronic devices, such as polymeric/organic light-emitting diodes (LEDs), field-effect transistors (FETs), photovoltaics (PVs), nonlinear optical devices, memory storage devices, energy storage photo detectors, and chemo- and bio-sensors [14,15,16,17,18,19]. Conjugated polymers with an interchanging sequence of suitable acceptor and donor units in the main chains can show a decrease in its band-gap energy owing to the interactions of the intramolecular charge transfer. Conjugated systems including double and single bonds were considered as a vital class of material for applications in optoelectronic devices owing to their π-excessive nature [20]. π-Conjugated polymers have been employed in extensive industrial applications [21].

Organic–inorganic hybrid solar cells (HSCs) have been broadly investigated as a substitute for complete OSCs. This solar cell type is indicated by mixing different inorganic semiconductors and organic semiconductors as the active layer of devices [22]. Polymer-based solar cells have been the topic of more emphasis and continuous study in recent decades, which have shown a substantial increase in PCEs from 6% to 17% [23,24,25,26,27,28,29]. Among them, organic solar cells containing small molecules and polymer-based solar cells have been studied broadly in industrial and academic communities owing to their large potential in substituting the conventional silicon-based solar cells as a low-cost solar energy. The first organic solar cell was reported by Tang et al. in 1986, which contained two layer photovoltaic cells prepared from copper phthalocyanine (CuPc) and a perylene tetracarboxylic derivative (PV) with a PCE of 1% [30]. A decade later, Heeger and colleagues reported the concept of a bulk hetero junction (BHJ) polymer solar cell using the blended system of conjugated polymer (donor) and fullerene (electron acceptor), in which the interfaces among the acceptor and donor offers pathways for charge generation, charge separation, and charge transportation [31,32].

The study of morphology and optical investigation of organic composite materials is vital prior to their applications in devices. The organic material performance can be modified by changing their optical properties and morphological and chemical structures [33]. Generally, in BHJ polymer solar cells, the active layer is composed of a low band gap conjugated polymer as the donor and fullerene/non-fullerene derivatives as an electron acceptor. Subsequently, with fast improvement in this field, current state-of-the-art materials have achieved PCEs of up to 12%, which is mostly due to the fast improvement of novel conjugated polymers with intelligent device optimization [34,35]. The light energy absorption by polymers in the UV-visible region involves electron transitions in n to π* from the ground state to higher energy states. This is because the absorption peaks for the transitions fall in an experimentally convenient region of the spectrum (200–700 nm). These transitions require an unsaturated group in the molecule to produce the pi electron [36]. Many of the optical transitions, which arise the existence of impurities, have energies in the visible region of the spectrum [37].

Noteworthy efforts have been made to narrow the polymer donor band gap so as to use the infrared photons, which caused the development of PCE from 5% to 12% in about a decade. Since 2017, the investigation of narrow-band-gap non-fullerene acceptors assisted in ushering in a novel era in OPV research and increased the attainability of PCE to 17% in just 3 years. In principle, the OPV boost seen in the last 15 years can be shortened with the effort to narrow the band gap of organic semiconductors and improve the position of the energy levels. There are many advantageous of a narrower band gap: (I) substantial infrared photons can be used, and thus the short circuit current density will increase considerably; (II) the energy offset of the lowest unoccupied molecular orbital energy levels or highest occupied molecular orbital energy levels between the donor and acceptor can be decreased, which can decrease the open-circuit voltage loss by reducing the loss resulting from the donor/acceptor charge transfer state; (III) owing to the unique molecular orbitals of organic semiconductors, the red-shifted absorption will encourage large transmittance in the visible area, which is ideal for the rear subcells in transparent OPVs and tandem-junction OPVs [38].

Global energy consumption is approaching 16 terawatts and is expected to triple by 2050. Solar cell research and development have been driven by this rapidly growing consumption of energy and also by rising concern regarding carbon emission from fossil fuel-based energy sources. Thus, PVs have remarkably drawn attention to technological improvement in recent years [22]. The most vital key to reducing the adverse impacts of conventional energy is to use clean and renewable energy that can contribute to the balance of global energy.

The low PCE of polymer fabrication hinders technologies based on renewable energy applications [39]. Therefore, further study is needed to reduce the band gap of conjugated polymers in order to collect a broader sunlight spectrum. Organic material stability is another issue that limits their applications in solar cells [40]. Most of the researchers have contributed to development of inexpensive and efficient inorganic or organic materials employed in photovoltaic devices or have aimed at OSC commercialization [41]. As stated by Kumar and Rao [3], the appropriate materials for suitable applications are those that have a band-gap energy (E_g_) in the range between 1.0 and 1.7 eV, such as mono-crystalline and polycrystalline silicon (E_g_ = 1.12 eV), CuInSe_2_ (E_g_ = 1.05 eV), and a-Si:H (E_g_ = 1.7 eV).

Lately, several organic solar cells with PCEs of more than 6% and up to 10% have been reported. Polymer tandem solar cells is a motivating area of research, and many attempts have been made to enhance the PCE of tandem organic solar cells on the basis of novel materials containing polymers [42]. The sunlight fraction absorbed is fixed for every material and changes with its chemical structure. The control of band gap and molecular energy states is vital for device performance. In this review paper, the synthesis of semiconductor polymers with different techniques is presented. Various applications based on conjugated polymers, including optoelectronic devices and organic solar cells, are discussed.

## 2. Electronic Structure and Doping in Conjugated Polymers

### 2.1. The Electronic Structure of Conjugated Polymers

In order to understand the electronic configuration of conjugated polymers, it is essential to compare the bond orientation and hybridization of saturated polymers and conjugated polymers. Polyethylene (**PE**) and polyacetylene (**PA**) are selected as examples of saturated and conjugated polymers, respectively.

In **PE**, each carbon atom in the main chain is sp^3^ hybridized, which is bonded with two neighboring carbon atoms and two hydrogen atoms to form four sigma (σ) bonds. Therefore, each carbon atom utilizes all four valence electrons, and these electrons are strongly localized. As a result, **PE** is electrically insulating, as there is no free electron in its backbone responsible for conducting electricity. Consequently, the optical band gap (E_g_) of **PE** is large and is around 8.0 eV [43]. In **PA**, each carbon atom is sp^2^ hybridized, which is bonded with two neighboring carbon atoms and one hydrogen atom to form three σ-bonds. The remaining unhybridized 2p_z_ orbital per carbon atom is perpendicular to the trigonal planar of the polymer backbone. Each unhybridized 2p_z_ orbital contains an unpaired electron and overlaps with the adjacent 2p_z_ orbital to form a π-bond, and ultimately they are delocalized along the entire polymer backbone. This electronic delocalization provides the semiconducting properties that permit the charge mobility along the conjugated polymer backbone [44].

The archetypal example of a conjugated polymer is **PA** (–CH–)_n_. If the carbon–carbon bonds in **PA** are equally long, the π-orbitals can be half-filled and it can possess a metallic behavior. It was predicted that this structure is unstable. In fact, the backbone of the **PA** has alternating bonds, in which there are slightly longer single and slightly shorter double bonds [45]. It has two isomeric forms, which are trans-polyacetylene (***t*-PA**) and cis-polyacetylene (***c*-PA**). According to the Peierls theory, each repeat unit in ***t*-PA** contains two carbon atoms (–CH=CH–)_n_. As a result, the π-band in ***t*-PA** is divided into two sub-bands, which are the filled π-band and empty π*-band. The difference in energy between these bands is the band gap (E_g_), and its value is 1.5 Ev [46].

### 2.2. Doping in Conjugated Polymers

Conjugated polymers in the pristine state are neutral and are usually semiconductors or insulators, which have low conductivities. Upon doping, charge carriers are created and move along the polymer chains. As a result, the conductivity remarkably increases by several orders of magnitude [47]. For instance, the conductivity of ***t*-PA** is lower than 10^−5^ S cm^−1^ in its undoped state. However, when it is doped with an oxidizing agent [48], the conductivity reaches a metallic region of ~10^3^ S cm^−1^.

Charge carriers can be generated via redox reactions. In conjugated polymers, the doping can be achieved in a number of different routes, for example photo doping, acid–base doping, charge injection, and redox doping.

**Figure 1:**
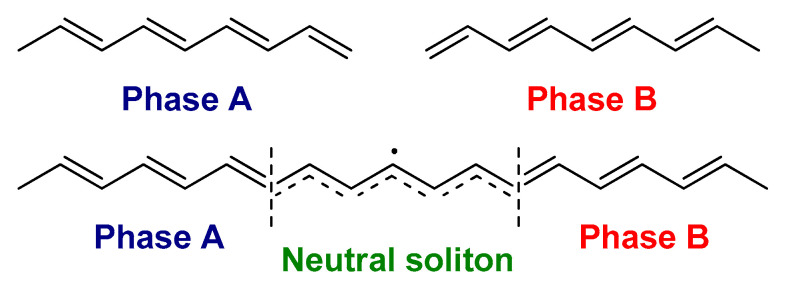
The schematic illustration of two degenerate ground state of the trans-polyacetylene (***t*-PA)** with domain boundary (neutral soliton) separating the two phases (A and B phases).

Among the conjugated polymers, ***t*-PA** type is unique since it has a degenerate ground state, i.e., two possible configurations (A and B phases) corresponding exactly to the identical total energy. The two phases are distinct from each other through interchanging the positions of single and double bonds [49]. As described above, each carbon atom in ***t*-PA** comprises an unpaired electron in the unhybridized 2p_z_ orbital. A neutral soliton (radical) is formed in pristine ***t*-PA,** with each chain having an odd number of carbons [50]. The soliton is a domain wall between the two phases (A and B phases) along one chain in ***t*-PA**, and it is spreading over several carbon atoms (approximately seven) (Figure 1) [44].

**Figure 2:**
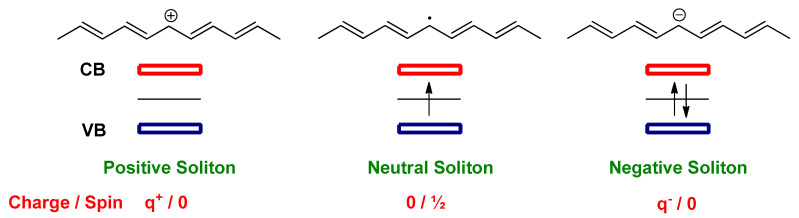
The schematic illustration of two degenerate ground state of the *t*-PA with domain boundary (neutral soliton) separating the two phases (A and B phases)**.**

A new localized energy level at mid-gap appears for soliton with respect to the valence band (VB) and conduction band (CB) levels of the ***t*-PA**. Once the soliton is neutral, the mid-gap is half-occupied. In the case of partially oxidized ***t*-PA** (p-type doping), a positively charged soliton is formed, and the mid-gap is empty. However, when ***t*-PA** is reduced partially (n-type doping), a negatively charged soliton is created, and the mid-gap contains two electrons. Soliton has a charge–spin relationships, since the neutral soliton has a half spin; however, charged solitons are spinless (Figure 2) [51].

**Figure 3:**
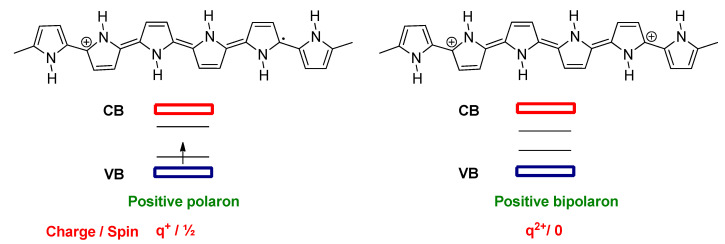
The schematic illustration of the chemical structures (upper), band structures, charges, and spins (lower) for a positive polaron and positive bipolaron in polypyrrole (**PPy)**.

Unlike ***t*-PA**, the other conjugated polymers such as ***c*-PA**, polypyrrole **(PPy)** and polythiophene **(PT)** possess two distinct resonance forms, namely aromatic and quinoid geometry, which are not energetically equivalent. The quinoid resonance structure has a higher energy than the aromatic resonance form. These types of polymers are called nondegenerate ground state conjugated polymers [52]. In these types of polymers, radical cations in p-type or radical anions in n-type doping are the main charged excitations. For example, in p-type doping of **PPy**, one electron is removed from the conjugated chain, which leads to the creation of a positively charged polaron (radical cation), and it is associated with a quinoid-like segment spreading over about four pyrrole rings. Two new electronic states in the gap are formed in polarons. For doped **PPy**, the states are about 0.5 eV far from the band edges [53]. The lower state in a positive polaron contains an unpaired electron and it has a spin of one half, while the second polaron is created by removing a second electron from the chain. The combination of two positive polarons causes the formation of energetically favorable dication species, which is called a positive bipolaron (Figure 3). In **PPy**, the bipolaron also spreads over four pyrrole rings, which is similar to the polaron [53]. In the bipolaron, the lower state is more upshifted from the VB, and the higher state is more downshifted from the CB with respect to the polaron states, and it is around 0.75 eV for **PPy** [53]. In positive bipolarons, those states are empty and are spinless. At higher doping levels, the bipolaron states can overlap to form bipolaron bands.

## 3. Methods for Preparation of Conjugated Polymers

Since the preparation of the first conjugated polymer (**PA**) in 1977, numerous methods for synthesis of conjugated polymers have been developed. In general, they can be classified into three main categories, namely the oxidative, the metal catalyzed cross-coupling, and the condensation polymerization methods.

### 3.1. The Oxidative Polymerizations

The oxidative polymerizations are subdivided into two categories: electrochemical oxidative, and chemical oxidative polymerizations.

#### 3.1.1. Electrochemical Oxidative Polymerizations

**Scheme 1:**
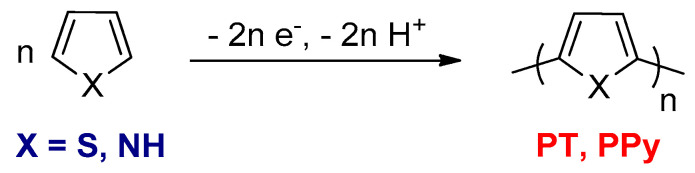
Synthesis of polythiophene (PT) or PPy by the electrochemical oxidative polymerizations.

This method is extensively used for the preparation of conjugated polymers such as **PPy** or **PT** from pyrrole or thiophene monomers, respectively (Scheme 1) [54]. In the electrochemical oxidative polymerization, the doped polymer film is directly deposited onto an electrode surface in a monomer solution that contains an electrolyte. The polymer film can be analyzed by electrochemical methods [55]. For this type of polymerization, an applied potential is required, and it must be higher than the oxidation potential of the corresponding monomers. The polymerization cannot be applied in large scale because of its limitation to the surface area of the electrode. 

**Scheme 2:**
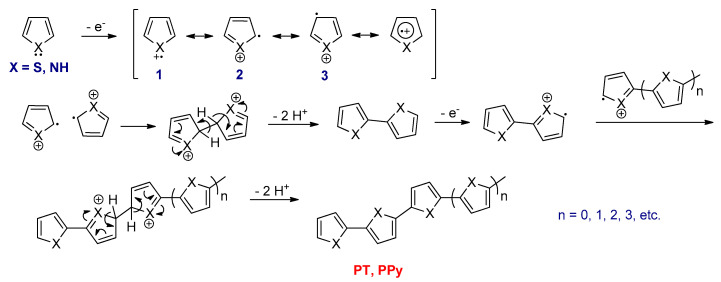
The mechanism of electrochemical oxidative polymerization for five membered heterocyclic monomers such as thiophene or pyrrole.

The mechanism of the electrochemical oxidative polymerization is electrochemical–chemical–electrochemical (E(CE)_n_) (Scheme 2). The first step involves the oxidation of the monomer to generate a radical cation [56]. This intermediate has three resonance forms (**1**, **2**, and **3**), while **2** is clearly the most stable one [57]. Then, two radical cations are dimerized at *α*-positions via radical–radical coupling to form a dihydro dication dimer. The next step is release of two protons to form dimer and the driving force of this step is returning the aromaticity of the dimer. This dimer is further oxidized to generate dimeric radical cation and can attack another radical to form a trimer after elimination of a proton. These reactions repeat until the polymer is formed [58].

#### 3.1.2. The Chemical Oxidative Polymerizations

**Scheme 3:**
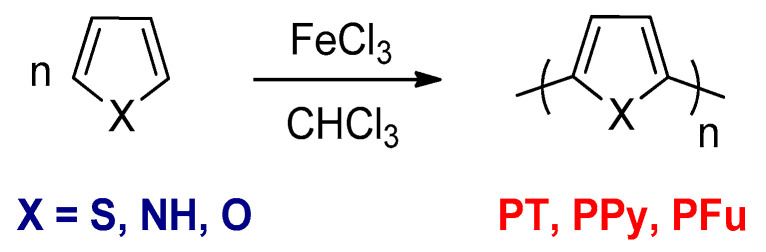
The synthesis of **PT**, **PPy**, and polyfuran (**PFu)** by the chemical oxidative polymerizations.

Some conjugated polymers can be prepared by chemical oxidative polymerizations, for instance, **PPy**, **PT**, and their derivatives such as poly(3-alkylthiophene)s (**P3ATs**) using anhydrous ferric chloride (FeCl_3_) or ammonium persulfate ((NH_4_)_2_S_2_O_8_) as oxidants [59]. FeCl_3_ has been widely used as an oxidizing agent in anhydrous chloroform. Yoshino and co-workers employed this type of polymerization for the synthesis of **PT**, **PPy**, and polyfuran (**PFu**) from the thiophene, pyrrole, and furan monomers, respectively (Scheme 3) [60].

**PT** is insoluble and has a lack of processability for optoelectronic applications. Introducing flexible side chains into the backbone of **PT** can dramatically increase the solubility of the **P3ATs**. **P3ATs** with straight alkyl side chains (butyl or longer) are soluble and processable [59]. 

**Figure 4:**
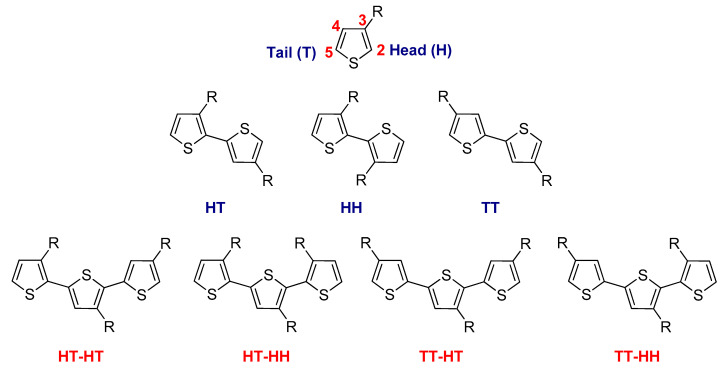
Possible regioisomeric couplings for 3-alkylthiophenes.

As 3-substituted thiophene monomers are asymmetric, coupling in the 2- and 5-positions of two 3-alkylthiophene units leads to three distinct regiochemical diads. The first coupling is head-to-tail (HT), a second coupling is head-to-head (HH), and the last coupling is tail-to-tail (TT). Moreover, when three 3-alkylthiophene monomers are linked together, the result is four possible triad regioisomers, which are HT–HT, HT–HH, TT–HT, and TT–HH (Figure 4). 

**Figure 5:**
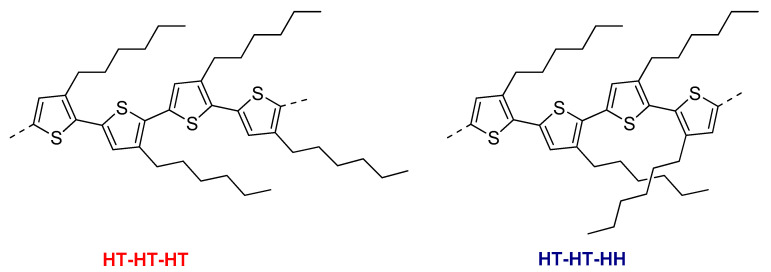
The schematic presentation of **rr-P3ATs** (**left**) and **ri-P3ATs** (**right**).

The incorporation of HH diad configurations leads to twisting of the conjugated polymer chain, due to the increased steric hindrance between the solubilizing alkyl chains and the lone electron pairs of adjacent sp^2^ sulfur atoms. **P3ATs** with a random mixture of HH linkages, TT linkages, and HT linkages are referred to as regioirregular (**ri-P3ATs**) (Figure 5). However, **P3ATs** that consist of only HT arrangements are referred to as regioregular head-to-tail (**rr-P3ATs**) (Figure 5). As a result, **ri-P3ATs** have lower effective conjugation lengths and larger bandgaps when compared to **rr-P3ATs** [2]. 

Generally, high molecular weight polymers and excellent yields can be achieved by chemical oxidative polymerization with FeCl_3_ as compared to the polymers synthesized by electrochemical oxidative polymerization. The polymerization of 3-alkylthiophenes either by electrochemical or chemical oxidative method gives **ri-P3ATs** with approximately 60–80% HT–HT couplings [61]. Andersson et al. reported that the chemical oxidative polymerization of a sterically hindered 3-(4-octylphenyl)thiophene by slow addition of FeCl_3,_ can provide regioregular poly (3-(4-octylphenyl) thiophene) (**POPT**) with up to 94% HT–HT couplings [62].

### 3.2. Transition Metal Catalyzed Cross-Coupling Polymerizations

**Scheme 4:**
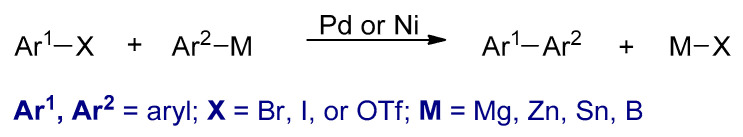
The transition metal catalyzed cross-coupling reactions.

Transition metal catalyzed cross-coupling polymerizations between organohalides (Ar^1^–X) and organometallic compounds (Ar^2^–M) are powerful synthetic methods for Csp^2^–Csp^2^ bond formation [63]. Several conjugated polymers could be synthesized by these reactions. The four most commonly used metal catalyzed cross coupling polymerizations are the Kumada–Corriu, Negishi, Stille, and Suzuki, in which different nucleophilic reagents such as aryl Grignard reagents, arylzinc reagents, aryl stannanes, and aryl boronic acids or esters are used, respectively (Scheme 4). In these reactions, palladium or nickel complexes are commonly used as catalysts. Furthermore, the metal catalysts that are used in these polymerizations are different, usually palladium-based catalysts are utilized in Stille and Suzuki. However, nickel-based catalysts are used in Kumada–Corriu. The polymers synthesized by these types of polymerizations are powders and are in their neutral states.

#### 3.2.1. Kumada–Corriu Cross-Coupling Reactions

**Scheme 5:**
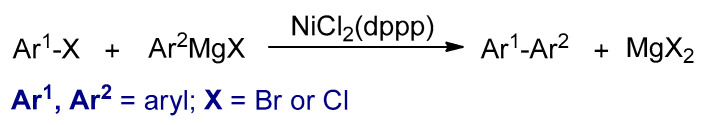
Kumada–Corriu cross-coupling reactions.

In 1972, Corriu et al. and Kumada et al. independently reported the coupling between Grignard reagents and aryl halides using nickel complexes as catalysts (Scheme 5) [64,65]. It was discovered that NiCl_2_ (dppp) was the most effective catalyst among all the nickel–phosphine complexes [65,66].

**Scheme 6:**
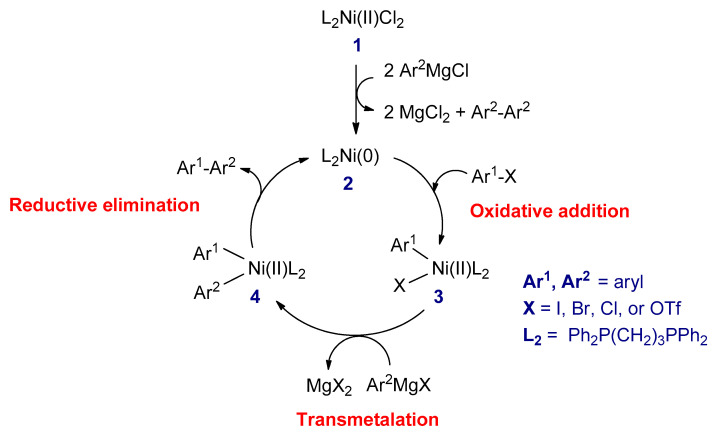
The catalytic cycle of the Kumada–Corriu reaction.

The proposed catalytic cycle of the Kumada–Corriu reaction is represented in Scheme 6. The first step is the reaction between dihalodiphosphinenickel (II) complex (**1**) with two equivalents of the Grignard reagent to form nickel(0) complex (**2**), which is the active catalyst in the catalytic cycle [64]. The second step is an oxidative addition of the active catalyst with an organohalide reagent to give halo(organo)nickel(II) complex (**3**). The third step is the transmetalation between **3** with the Grignard reagent to generate a new diorgano nickel(II) complex (**4**). In the final step, the latter complex undergoes the reductive elimination to form the coupled product (Ar^1^–Ar^2^) and concomitantly regenerate the L_2_Ni(0) complex to complete the catalytic cycle [67]. 

**Scheme 7:**
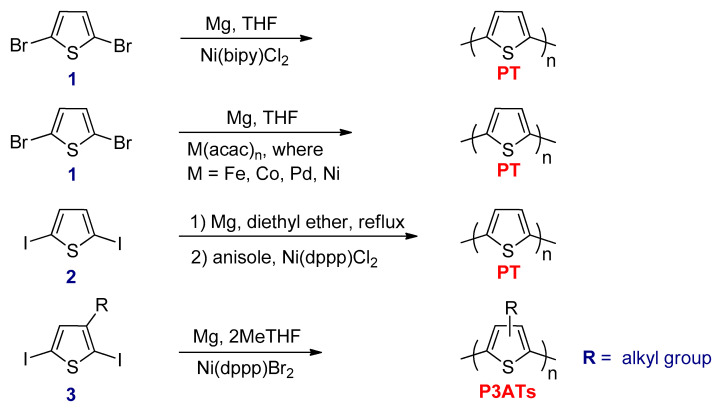
The synthesis of **PT** and **P3ATs** by Kumada–Corriu coupling reaction.

In 1980, the synthesis of **PT** was published by two research groups using Kumada–Corriu coupling reaction. The first group was Yamamoto, in which **PT** was synthesized by treating 2,5-dibromothiophene (**1**) with magnesium metal in tetrahydrofuran (THF) using Ni(bipy)Cl_2_ as a catalyst (Scheme 7) [68]. Lin and Dudek were the second group that treated same starting material as used by Yamamoto et al. in the presence of various transition metal acetylacetonate complexes (M(acac)_n_) (Scheme 7) [69]. The **PT** prepared by these research groups had low molecular weights and were insoluble, even in THF. Soon after, Wudl et al. reported the synthesis of **PT** by reacting 2,5-diiodothiophene (**2**) with magnesium metal in diethyl ether to form 2-iodo-5-iodomagnesiothiophene, which is the Grignard reagent intermediate [70]. This intermediate was separated and treated with hot anisole, and then Ni(dppp)Cl_2_ was added as a catalyst (Scheme 7).

The first chemical preparation of soluble **P3ATs** was synthesized by Elsenbaumer and co-workers via the Kumada–Corriu coupling reaction [71]. The 3-alkylsubstituted-2,5-diiodothiophene (**3**) was treated with one equivalent of magnesium metal in THF (or 2MeTHF) as a solvent for the reaction to form the Grignard reagent (Scheme 7) [72]. Ni(dppp)Br_2_ was used as a catalyst and subsequently added to the resulting Grignard reagent to form soluble **P3ATs** with low number average molecular weight (*M*_n_ = 3000–8000 g mol^−1^). The **P3ATs** synthesized by this method contained only 50–80% HT–HT couplings, and regioregularity could not be controlled.

**Scheme 8:**
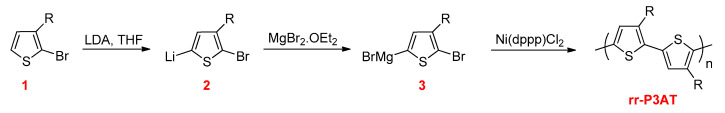
The synthesis of **rr-P3ATs** by the McCullough method.

The first synthesis of **rr-P3ATs** was developed by McCullough and Lowe in 1992 (Scheme 8) [73]. This method starts with selective lithiation at the 5-position of 2-bromo-3-alkylthiophene (**1**) with lithium diisopropylamide (LDA) at cryogenic temperature to generate organolithium intermediate (**2**), which is stable at this temperature with only 1–2% of metal–halogen exchange [74]. This intermediate is changed to the organomagnesium intermediate (**3**) by the addition of magnesium bromide ethyl etherate (MgBr_2_.OEt_2_) and subsequently polymerized in situ using the Kumada–Corriu cross-coupling reaction by adding Ni(dppp)Cl_2_ catalyst to give **rr-P3ATs** with almost 100% of HT–HT couplings [75].

**Scheme 9:**
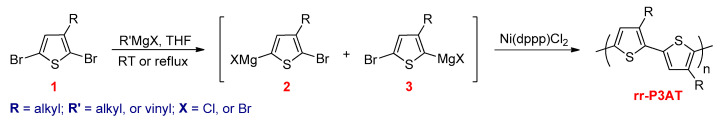
The synthesis of **rr-P3ATs** by the grignard metathesis (GRIM) route.

Later on, McCullough and co-workers prepared **rr-P3ATs** via the Grignard metathesis (GRIM) method (Scheme 9) [76]. Treatment of 2,5-dibromo-3-alkylthiophene (**1**) with one equivalent of commercially available Grignard reagent resulted in a mixture of two regiochemical isomers (**2** and **3**) in a ratio of 85:15. This ratio was found independently of the type of the Grignard reagent, temperature, and reaction time used. The Ni(dppp)Cl_2_ as a catalyst was added to the above mixture, yielding **rr-P3ATs**, which contained more than 99% HT–HT couplings. This process does not require cryogenic temperature, which is necessary in the McCullough route. Moreover, this method does not require the use of highly reactive metals and high molecular weight **rr-P3ATs**, and it can be prepared in the kilogram scale [77]. The reason provided for the regioregularity of the polymers obtained is that intermediates **2** and **3** are each involved separately in the growth of polymer chains, which leads to selective formation of head-to-tail **P3ATs**. 

#### 3.2.2. Negishi Cross-Coupling Reactions

**Scheme 10:**
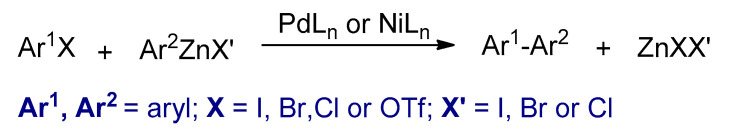
Negishi cross coupling reactions.

Negishi reported the first coupling between organozinc derivatives and organohalides by using Ni or Pd as the catalyst (Scheme 10) [78].

**Scheme 11:**
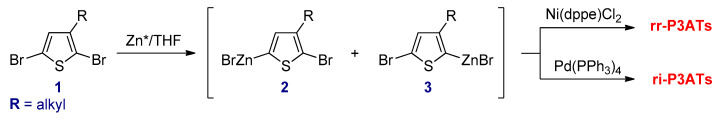
Chen and Rieke method for synthesis of **rr-P3ATs** and **ri-P3ATs**.

Chen and Rieke reported the synthesis of **rr-P3ATs** via the Negishi cross-coupling reaction (Scheme 11) [79]. In this method, 2,5-dibromo-3-alkylthiophene (**1**) was reacted with Rieke zinc (Zn*) at cryogenic temperature resulting in a mixture of the two regioisomers (**2**) as the predominant intermediates and **3** in minor quantities. A regioselectivity was achieved as high as 97–98% at −78 °C for most cases. In addition, the ratio between these isomers depends less on the length of the alkyl chains [80]. These organozinc intermediates in situ undergo regioselective polymerization by adding a catalytic amount of Ni(dppe)Cl_2_ to afford the 97–98% head-to-tail **rr-P3ATs**, whereas use of a Pd(PPh_3_)_4_ as a catalyst under identical conditions completely yields **ri-P3ATs**.

#### 3.2.3. Stille Cross-Coupling Reactions

**Scheme 12:**
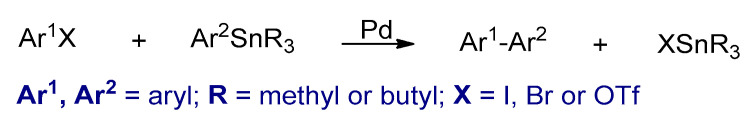
The Stille cross coupling reactions.

The coupling reaction of organic electrophiles such as aryl halides or triflates and aryl stannanes catalyzed by palladium is called the Stille coupling reaction, and it is one of the most selective and powerful synthetic approaches to form Csp^2^–Csp^2^ bonds (Scheme 12) [81,82]. 

One of the advantages of the Stille reaction are the use of organotin reagents that can be prepared easily. They are also stable toward air/moisture, and the reaction is accomplished under neutral conditions. Another advantage of this type of reaction is that they tolerate different kinds of functional groups on both partners, including ester or other sensitive groups such as nitrile and alcohol without any protection/deprotection strategies [83]. Conversely, it has some disadvantages, for example, organotin compounds and their by-products are toxic materials [84,85].

**Scheme 13:**
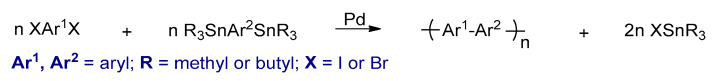
The Stille polycondensation.

A wide variety of different conjugated polymers with high molecular weights have been synthesized by using palladium-catalyzed Stille polycondensation between electron-rich distannane monomers and electron-deficient organodihalide monomers (Scheme 13) [81,86]. 

**Scheme 14:**
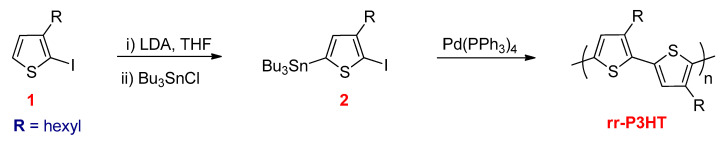
The synthesis of **rr-P3HT** by Stille polycondensation.

Iraqi and Barker synthesized regioregular poly(3-hexylthiophene) (**rr-P3HT**) through the Stille polycondensation using 2-iodo-3-hexyl-5-*n*-butylstannylthiophene monomer (**2**) (Scheme 14) [87]. This monomer was synthesized by cryogenic lithiation of 2-iodo-3-hexylthiophene (**1**) at the 5-position using LDA and subsequently treated with tri-*n*-butyltin chloride. The monomer undergoes homopolymerization using a variety of solvents, which results in **rr-P3HT** with greater than 96% HT–HT couplings. 

**Scheme 15:**
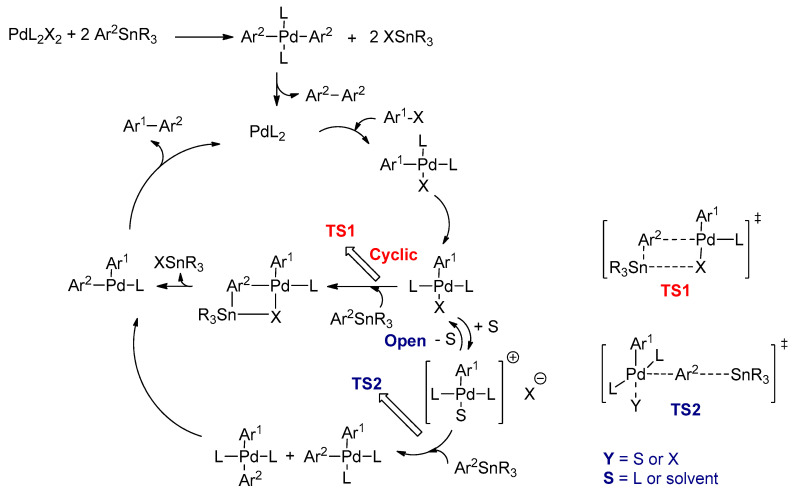
The catalytic cycle of Stille cross-coupling reaction.

The accepted catalytic cycle for the Stille reaction is illustrated in Scheme 15 [82]. The reaction consists of four main steps: (1) oxidative addition; (2) transmetalation; (3) trans to cis isomerization; and finally (4) reductive elimination. The Pd^(0)^, such as Pd(PPh_3_)_4_ and Pd_2_(dba)_3_, or Pd^(II)^, such as Pd(OAc)_2_ and Pd(PPh_3_)_2_Cl_2_, are used as the catalysts. In the beginning of the catalytic cycle, the Pd^(II)^ change into to Pd^(0)^L_2_, which is activated by arylstannane monomer and allows access into the catalytic cycle. The first step is the oxidative addition of an organic electrophile (Ar^1^X) to the Pd^(0)^L_2_ to form Ar^1^Pd^(II)^L_2_X intermediate. Espinet and Casado found that this complex is formed in the cis-configuration, and then it is isomerized to the more stable trans-configuration [88]. The mechanistic details of the transmetalation step were published by Espinet and Echavarren, who proposed cyclic and open pathways [89]. In the cyclic pathway, trans-Ar^1^Pd^(II)^L_2_X intermediate undergoes associative L-for-Ar^2^ substitution through transition state (**TS1**), in which the Sn and Pd metals are bridged by X and Ar^2^. As a result, a T-shaped cis-Ar^1^Ar^2^Pd^(II)^L complex is formed after elimination of XSnR_3_; the organic product (Ar^1^–Ar^2^) is then generated, and the final step is called reductive elimination [90]. The open pathway is favored, since no bridging ligands are available, and in highly polar solvents/weakly coordinating anion (like triflate), in which trans-Ar^1^Pd^(II)^L_2_X is readily substituted by the ligand or solvent [91]. In this pathway, X is replaced by coordinating solvent (S) or ligand (L) to form competitively cis- and trans-Pd^(II)^L_2_Ar^1^Ar^2^ complexes through the transition state (**TS2**) [92]. Finally, the coupled product is formed.

#### 3.2.4. The Suzuki–Miyaura Reactions

**Scheme 16:**
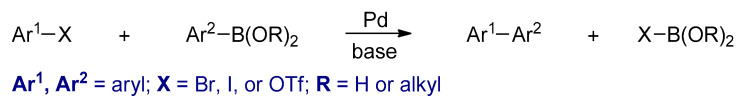
The Suzuki–Miyaura cross coupling reaction.

The Suzuki–Miyaura reaction has become one of the most important and general methodologies for the construction of carbon–carbon bonds [93]. This reaction is comprised of the coupling between different kinds of organic electrophiles such as halides or triflates and organoboronic acids or esters in the presence of base and palladium complexes (Scheme 16) [94].

The Suzuki–Miyaura reaction offers several advantages, such as commercial availability of the reagents and mild reaction conditions. Organoboron compounds are generally inert toward oxygen/water and thermally stable [95]. Moreover, they can tolerate various types of functional groups. The by-products of organoboron compounds are non-toxic and can be easily separated [96]. Therefore, this reaction is not only suitable for laboratories but also appropriate for large scale synthesis. 

**Scheme 17:**
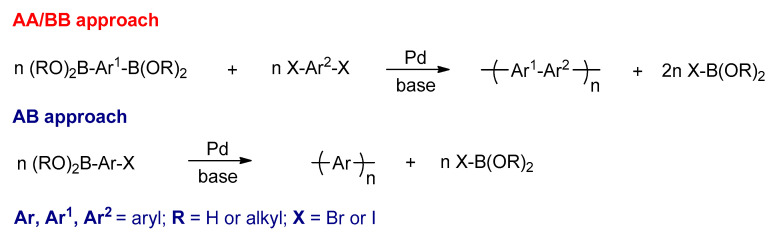
The Suzuki polycondensation.

The Suzuki–Miyaura reaction has been used for preparing various types of conjugated polymers, for example, poly(arylene)s and their analogues. The Suzuki polycondensation (**SPC**) reaction is a step growth polymerization between two different monomers in the AA/BB approach, in which one of the aromatic monomers is carrying two boronic acids (or esters) and the second monomer has two halogens (bromides or iodides) to form alternating copolymers [97]. The **SPC** can also use a bifunctional (AB) monomer (AB approach), in which a monomer carries boronic acid (or ester) on one side and the halogen on the other side to form homopolymers (Scheme 17) [97]. 

**Scheme 18:**
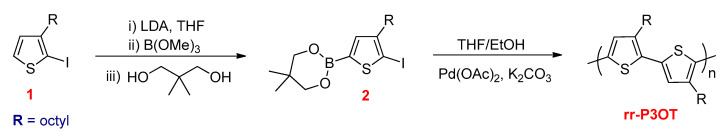
The synthesis of **rr-P3OT** by the suzuki polycondensation (**SPC)**.

Guillerez and Bidan reported the synthesis of a regioregular poly(3-octylthiophene) (**rr-P3OT**) through the **SPC** using 2-iodo-3-octyl-5-boronatothiophene monomer (**2**) [98]. Similar to the Stille reaction, this monomer was obtained from 2-iodo-3-octylthiophene (**1**) by selective lithiation at 5-position at −40 °C using LDA and subsequently treated with organoboron reagent. The polymerization was carried out by coupling (**2**) using Pd(OAc)_2_ as a catalyst, yielding **rr-P3OT**, which contained 96–97% HT–HT couplings (Scheme 18). 

**Scheme 19:**
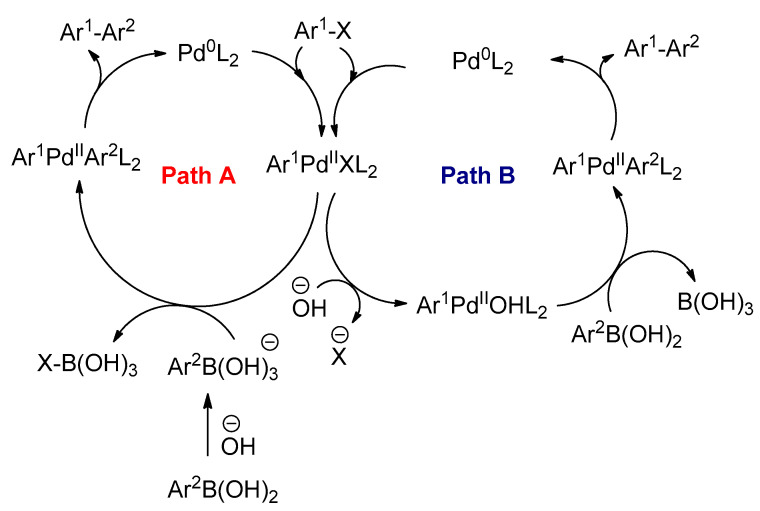
The catalytic cycle of the Suzuki–Miyaura cross-coupling reaction.

The catalytic cycle is similar to that of common cross-coupling reactions, and it takes place in three steps: (1) oxidative addition; (2) transmetalation, and (3) reductive elimination (Scheme 19) [95]. The oxidative addition of aryl halides (Ar^1^X) to the Pd^0^L_2_ complex is the first step of the catalytic cycle [99]. In this step, palladium oxidizes from an oxidation state of (0) to (+2) to form the Ar^1^Pd^II^XL_2_ complex. This complex is formed in a square planar cis-configuration, and afterward it isomerizes to a more stable trans-configuration, which is the first isomerization in the catalytic cycle [100]. These results were also confirmed by Braga and co-workers [101]. Although the oxidative addition and reductive elimination steps have been studied experimentally and theoretically, the mechanism of these two steps is well understood, and they are common in all transition metal catalyzed cross-coupling reactions of organometallics. The transmetalation step is different from one catalytic cycle to another [102]. This step is highly dependent on the type of organometallic reagent used or the reaction conditions for the couplings [103]. The base such as sodium hydroxide is required for the transmetalation step in the Suzuki–Miyaura cross-coupling reaction [104]. Two pathways are generally proposed for transmetalation (Scheme 19). In path A, the base reacts with an organoboronic acid to form a negatively charged boronate (Ar^2^B(OH)_3_^−^). This increases the nucleophilicity of the aryl group on the boron atom, and it can undergo transmetalation with the Ar^1^Pd^II^XL_2_ complex [95]. Alternatively, in path B, the halogen in the Ar^1^Pd^II^XL_2_ complex is replaced by a negatively charged base OH^−^ or OR^−^ depending on the type of the base used. Oxo-palladium(II) complexes (Ar^1^Pd^II^OHL_2_ or Ar^1^Pd^II^ORL_2_) are formed, and subsequently they can react with neutral organoboronic acids [105,106]. 

Hartwig et al. studied the kinetics of the transmetalation step in the Suzuki–Miyaura cross-coupling reaction [105]. It was found that the rate of transmetalation between boronate (Ar^2^B(OH)_3_^−^) and Ar^1^Pd^II^XL_2_ complex was around four orders of magnitude slower than that between organoboronic acid and Ar^1^Pd^II^OHL_2_. In addition, electrospray ionization mass spectrometry (ESI–MS) was utilized by Aliprantis and Canary to detect the intermediates in the Suzuki–Miyaura coupling reaction [107]. In their studies, two intermediates such as [Ar^1^Pd(PPh_3_)_2_Br]^+^ and Ar^1^PdAr^2^(PPh_3_)_2_ were formed. However, oxo-palladium intermediates such as Ar^1^PdOH(PPh_3_)_2_ or Ar^1^PdOCH_3_(PPh_3_)_2_ were not observed. 

The final step of the catalytic cycle is the reductive elimination [108]. Ar^1^Pd^II^Ar^2^L_2_ is formed in the trans-configuration as a result of the transmetalation process. Therefore, it isomerizes to cis-configuration, and coupling product (Ar^1^–Ar^2^) is obtained.

#### 3.2.5. Direct Hetero (Arylation) Cross Coupling Reactions

**Scheme 20:**
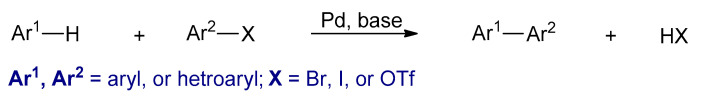
The direct hetero (arylation) reaction.

The formation of carbon–carbon bonds between (hetero) aryl halides or pseudohalides and unfunctionalized (hetero) arenes to form hetero–hetero or biaryl molecules via palladium-catalyzed direct hetero (arylation) has attracted significant attention (Scheme 20) [109]. 

This novel reaction has recently emerged as an economically-preferable and environmentally-friendly alternative to traditional cross-coupling reactions [110]. As the latest developments in organic and polymer synthesis, these reactions have numerous advantageous over conventional metal-catalyzed cross-coupling reactions; for instance, (1) they do not require organometallic monomers, which are in some cases difficult to purify; (2) the byproduct is HX, which is less toxic than organotin compounds (Me_3_SnX or Bu_3_SnX) in the Stille reactions; (3) they have fewer synthetic steps; (4) they have lower cost; and (5) in some cases they have higher yields [111].

**Scheme 21:**
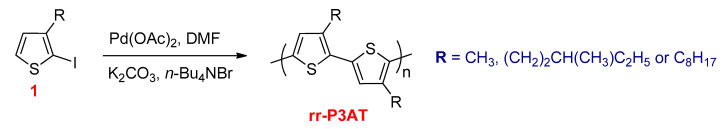
The synthesis of **rr-P3AT** via direct heteroarylation polymerization (**DHAP)**.

**P3AT** was the first conjugated polymer synthesized by Lemaire and co-workers using direct heteroarylation polymerization **(DHAP)** [112]. The dehydrohalogenative polycondensation of iodinated thiophene derivative (**1**) using Pd(OAc)_2_ as a catalyst, K_2_CO_3_ base, and stoichiometric amounts of tetra-*n*-butylammonium bromide (*n*-Bu_4_NBr) gave **rr-P3AT** with low molecular weight (*M*_n_ ~3000 g mol^−1^) (Scheme 21). 

**Scheme 22:**
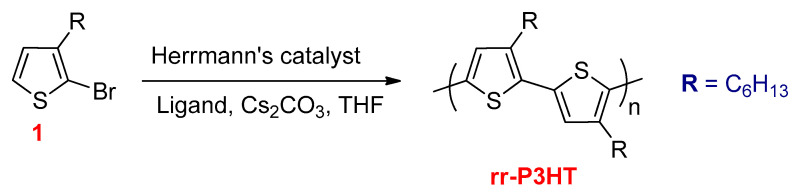
The synthesis of **rr-P3HT** via **DHAP**.

Ozawa and co-workers synthesized higher molecular weight **P3HT** (*M*_n_ up to 30,000 g mol^−1^) with high regioregularity of 98% from 2-bromo-3-hexylthiophene (**1**) using Herrmann^’^s catalyst and P(C_6_H_4_-*o*-NMe_2_)_3_ as a ligand (Scheme 22) [113].

**Scheme 23:**
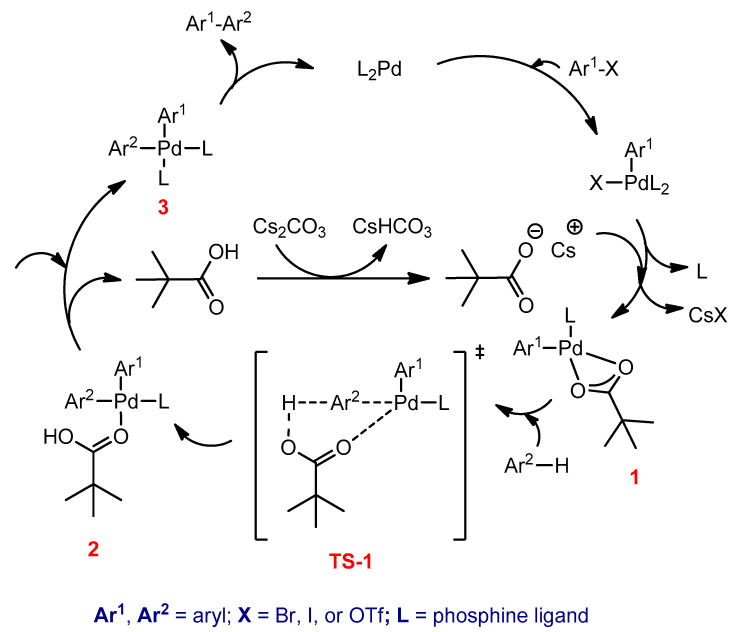
The catalytic cycle of the **DHAP**.

Concerted metalation–deprotonation (CMD) is the most recent proposed mechanistic pathway for the Pd-catalyzed direct arylation reactions [114,115,116]. The catalytic cycle for coupling of aryl halide (Ar^1^–X) and arene (Ar^2^–H) using the Pd-phosphine catalytic system, stoichiometric amounts of pivalic acid as an additive, and cesium carbonate base is illustrated in Scheme 23 [117]. The first step in the catalytic cycle is the oxidative addition, which is similar to that of the Stille and Suzuki coupling reactions. In the second step, a halogen atom and one phosphine ligand are replaced by pivalate anion to form complex **1**. The pivalate anion functions as a proton shuttle in this complex, which abstracts a proton from Ar^2^–H, and concurrently Pd–C bond is formed via a transition state (**TS-1**). This transition state is transformed to biaryl palladium complex (**2**) and is followed by the exchange of pivalic acid by phosphine ligand to create biaryl palladium diphosphine complex (**3**). Finally, the coupled product (Ar^1^–Ar^2^) is formed from the latter complex, and the palladium catalyst is regenerated and re-enters the catalytic cycle [118]. 

### 3.3. Yamamoto Coupling Reactions

**Scheme 24:**
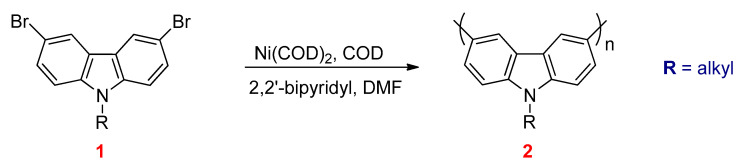
The synthesis of polycarbazoles by Yamamoto reaction.

A variety of conjugated polymers such as polyfluorenes, polyphenylenes, polycarbazoles, and polythiophenes were prepared by Yamamoto coupling reactions [119]. The reaction is carried out through dehalogenation polycondensation of dihalide monomer in the presence of zero-valent nickel complex such as Ni(COD)_2_ as a catalyst and COD as a neutral ligand. Poly(*N*-alkyl-3,6-carbazole)s (**2**) were successfully synthesized by this method from *N*-alkyl-3,6-dibromocarbazoles (**1**) as starting materials (Scheme 24) [120]. 

The resulting polymers have high molecular weights. The main drawbacks of the Yamamoto coupling reaction are the stoichiometric amounts of the catalyst that must be taken into consideration, and the catalysts are instable.

### 3.4. Condensation Polymerization Methods

**Scheme 25:**
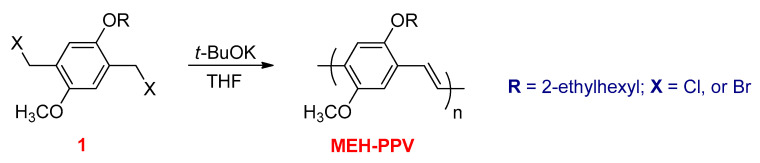
Gilch method for preparation of **MEH–PPV**.

These types of polymerizations have been widely used for synthesi**z**ing arylenevinylene polymers and copolymers. Poly(*p*-phenylene vinylene) (**PPV**) and its derivatives poly[2-methoxy-5-(2-ethylhexyloxy)-1,4-phenylenevinylene] (**MEH–PPV**) and poly[2-methoxy-5-(3′,7′-dimethyloctyloxy)-1,4-phenylenevinylene] (**MDMO–PPV**) can be prepared by condensation polymerizations. Wessling and Gilch are the two most common types for condensation polymerizations. To date, the ultimate versatile route for the synthesis of dialkoxy-substituted **PPV** derivatives is known as the Gilch method. The most extensively studied **PPV** derivative is **MEH–PPV**, which has been synthesized by this method [121]. The 1,4-dihalo-2,5-dialkoxy-*p*-xylene (**1**) is polymerized with a large excess of base, such as potassium *tert*-butoxide (*t*-BuOK), in THF (Scheme 25).

## 4. Applications of Conjugated Polymers

Organic semiconducting materials are a promising alternate to inorganic semiconducting materials. The development of organic semiconductors has potential applications in optoelectronic devices like light-emitting diodes (LEDs) [122,123], field effect transistors (FETs) [124,125], and photovoltaics (PVs) [126].

### 4.1. Organic Light Emitting Diodes (OLEDs)

**Figure 6:**
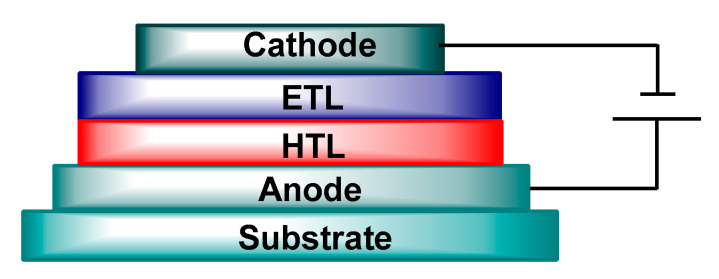
A typical bilayer organic light emitting diode (OLED).

Electroluminescence was discovered for anthracene by Pope et al. in 1963 [127]. In 1987, Tang and VanSlyke demonstrated an effective electroluminescence in organic small molecules in a bilayer device with 1% external quantum efficiency [128]. In 1990, Burroughes et al. developed the first OLED using a semiconducting polymer such as **PPV**, which is called polymer light-emitting diode (PLED) [123]. The typical OLED comprises of one or more organic layers sandwiched between two electrodes [129]. A schematic illustration of a typical bilayer OLED is demonstrated in Figure 6. Indium tin oxide (ITO), which is a transparent conducting oxide (TCO), is coated on glass substrate or flexible polymer. It is frequently used as the anode and it has a high work function. Low work function metals like calcium, magnesium, or aluminum are commonly utilized as the cathode [130]. In OLED, one of the organic layers is a hole transport layer (HTL), the other one is an electron transport layer (ETL), and one of the two layers must be emissive. HTL has low ionization potential (IP) but ETL has high electron affinity (EA) (Figure 6).

**Figure 7:**
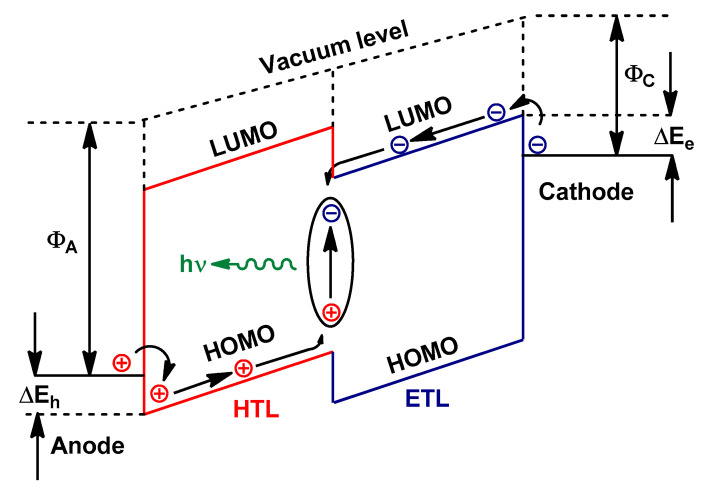
The energy level diagram and the operation of two-layer OLED. Φ_c_ and Φ_A_ are abbreviations of the work functions of the cathode and anode electrodes, respectively. ΔE_e_ and ΔE_h_ are the electron and the hole injection barriers, respectively.

The operation process of a two layer OLED is illustrated in Figure 7. When a forward voltage is applied across the device, electrons and holes are injected from the cathode and anode into the LUMO and HOMO of ETL and HTL, respectively. Next, they are transported into the HTL/ETL interface and recombine to form excitons. Subsequently, they may decay radiatively to produce light emission [131].

OSCs have shown great promise as cheap photovoltaic devices for solar energy conversion over the last decade. Interfacial engineering offers a strong strategy to improve efficiency and stability of OSCs. With the fast advances of active layer materials and interface layer materials, PCEs of both single-junction and tandem OSCs have increased a landmark value of 10%. Electron or hole transporting materials containing polymers/small-molecules, metal oxides, carbon-based materials, metals and metal salts/complexes, organic–inorganic hybrids/composites, and other emerging materials are systemically presented as cathode and anode interface layers for high performance OSCs. Moreover, incorporating these hole-transporting and electron-transporting layer materials as building blocks, a variety of interconnecting layers for traditional or inverted tandem OSCs have been documented for achieving high performance OSCs [132].

A variety of conjugated polymers have been investigated for light emitting diode applications, such as poly(*p*-phenylene) (**PPP**) [133], polythiophenes (**PT**)s [134,135], polycarbazoles (**PCz**)s [136], and polyfluorenes (**PF**)s [137]. The color of emission depends upon the band gap of the conjugated polymer light emitting materials, which emit light from ultraviolet to near infrared.

### 4.2. Organic Field Effect Transistors (OFETs)

**Figure 8:**
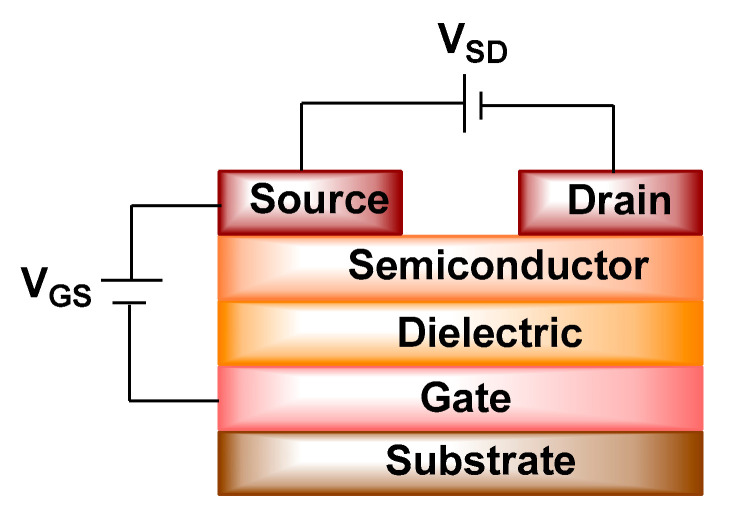
The organic field effect transistor (OFET) device configuration.

Typical OFETs are composed of a gate electrode, insulating layer, organic semiconducting layer, and the source and drain electrodes (Figure 8) [138]. When a sufficient gate-source voltage (V_GS_) is applied between gate and source electrodes, charge carriers are formed and accumulated at the organic semiconducting/dielectric interface. As a result, a channel is generated and by applying a source-drain voltage (V_SD_), these charges are transported and a current flows from source to drain electrode. This state of the OFET device is called on, while when V_GS_ = 0, OFET is termed off [124].

The types of the charge carriers formed in OFETs depend on the sign of V_GS_. When negative potential (V_GS_ ˂ 0) is applied, the holes are generated, and the type of organic semiconductor is called p-type. However, when positive voltage is biased (V_GS_ ˃ 0) the electrons are formed and the type of organic semiconductor is designated as n-type. In addition, in a few cases, organic semiconductors are able to transport both electrons and holes, which are considered as ambipolar [139].

Three kinds of π-conjugated materials have been used in OFETs, namely small molecules [16], oligomers [140], and polymers [16]. They should have good stability, high charge carrier mobility, and low cost for production and device fabrication.

Polythiophenes are frequently utilized in OFET devices. For the first time, unsubstituted polythiophene (**PT**) was used as semiconducting material in a polymer field effect transistor (PFET) by Ando and co-workers [141]. It showed a charge carrier mobility of 10^−5^ cm^2^ V^−1^ s^−1^. **rr-P3HT** exhibited significantly higher hole mobility compared to **ri-P3HT** (Figure 5) [142,143,144].

### 4.3. Organic Photovoltaics (OPVs)

At present, the majority of global energy consumption originates from fossil fuels [145]. Burning fossil fuels releases greenhouse gases such as CO_2_, which has an adverse impact on the environment and causes air pollution, global warming, and climate change [146]. In addition, the stock of these non-renewable energy sources is limited and cannot provide enough energy when the world’s population will increase. To tackle these issues, renewable energy sources such as hydropower, bioenergy, wind power, and solar and geothermal energy have been developed in the past few decades. Harvesting solar energy and converting it into electricity via photovoltaic (PV) technology is a promising solution to growing energy demand [147]. The power of the sunlight that strikes the surface of the earth amounts to 165,000 terawatt (TW) per day, and its energy of one hour is enough to provide the global energy consumption in an entire year [148,149,150].

The first inorganic crystalline silicon solar cell with efficiency of 6% was reported in 1954 by Chapin and co-workers [151]. Currently, single junction crystalline silicon solar cells dominate the photovoltaic technology and have reached efficiencies up to 25% [152,153]. The indirect band gap of silicon makes silicon-based solar cells necessitate relatively thick active layers to absorb sufficient sunlight [154]. In addition, high purity silicon crystals are required to avoid recombination losses during charge carrier transportation and collection. Furthermore, the manufacturing process requires high energy, which combined with the high cost of silicon, makes the silicon-based solar cell expensive. Therefore, silicon-based photovoltaics provide a small amount of the global energy production.

Thus, alternative semiconducting materials have emerged during the last couple of decades in order to reduce the materials costs. Copper indium gallium diselenide (**CIGS**), cadmium telluride (**CdTe**), and amorphous and nano-crystalline silicon (**a-Si** and **nc-Si**) are candidates for thin film photovoltaic devices. These inorganic materials are direct absorbers, and they can absorb more photons than crystalline silicon [155]. Amorphous and nano-crystalline silicon deliver a power conversion efficiency(PCE) of 10.1% [156]. However, higher efficiencies of 16.5 and 21.5% have been reported for **CdTe** and **CIGS**, respectively [157,158]. Although, these kinds of solar cells have shown decent efficiencies, the material availability, toxicity (Cd, Te), and difficulties in controlling large scale uniform films are obstacles for widespread commercialization.

Organic photovoltaic technology generally includes small molecules [159,160], conjugated polymers [161,162], and dye-sensitized [163,164]-based solar cells. In particular, polymer photovoltaics have received substantial interest because of a number of reasons such as low cost, easy processability, mechanical flexibility, lightweight, and large scale roll-to-roll (R2R) production [165,166,167]. In addition, the optoelectronic properties of the conjugated polymers could be adjusted by molecular design [168]. Furthermore, they have high absorption coefficients, and therefore only 100–200 nm active layer thickness is required for adequate absorption of sunlight [169]. Although PSCs have advanced very rapidly, their power conversion efficiencies and lifetime are still inferior compared to inorganic solar cells [170,171].

## 5. Architecture of Polymer Solar Cells

### 5.1. Single Layer

Single layer cells are the simplest device structure of organic photovoltaics, and they contain only one organic layer between two electrodes [172]. The anode is made of an ITO. The cathode is composed of a metal such as calcium, magnesium, or aluminum. The difference in the electrode work function provides a built-in electric field, which is not high enough to overcome the exciton binding energy, which is larger than 0.5 eV in conjugated polymers [173]. This energy is of several orders of magnitude, which is higher than that of thermal energy (*kT* (300 K) = 0.026 eV), and therefore, in such devices, the electric fields are insufficient to separate the excitons into free electrons and holes [155]. **PPV** was used in single layer photovoltaic cells and provided very low external quantum efficiency (EQE) in the order of 1% with a PCE lower than 0.1% [174].

### 5.2. Bilayer Planar Heterojunction

In a two layer planar heterojunction, acceptor and donor layers are sandwiched between the electrodes. The first two layer planar photovoltaic cell was developed by Tang, in which two different organic semiconductors, a donor (D) and an acceptor (A), were embedded between a transparent ITO and a semitransparent metal electrode [30]. Copper phthalocyanine (CuPc) was used as a D material, and a perylene tetracarboxylic derivative (PV) was used as an A material. Tang’s device achieved a PCE of about 1% under simulated air mass 2 (AM2) conditions. The overall improvement efficiency in bilayer devices is mainly due to the exciton dissociation at the D–A junction, which is much more efficient than the polymer/electrode junction in single layer devices [175,176].

The performance of bilayer devices is greatly restricted by the exciton diffusion length [177]. For most conjugated polymers, the exciton diffusion length is 4–20 nm [178,179,180]. Consequently, only the excitons that are produced near the D–A junction can be dissociated. The majority of excitons created far from the interface are lost by recombination, which leads to the low quantum efficiency and diminished solar cell performance [17].

**Figure 9:**
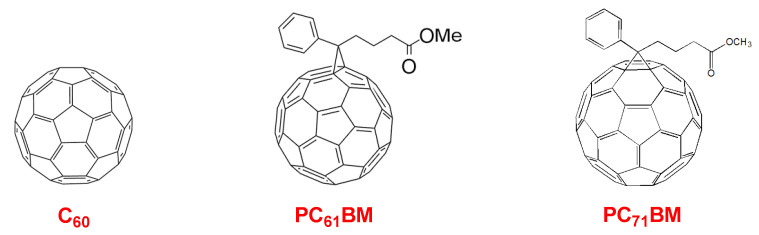
The molecular structure of C_60_ and its derivatives.

One of the major breakthroughs in the field of solar cell technology was the replacement of n-type material by Buckminsterfullerene (C_60_) and its derivatives, such as [6,6]-phenyl-C_61_-butyric acid methyl ester(**PC_61_BM**) and [6,6]-phenyl-C_71_-butyric acid methyl ester(**PC_71_BM**), in organic photovoltaic (OPV) devices (Figure 9).

Fullerenes have become standard acceptors for organic solar cells due to their various advantages. First, they have deep-lying LUMO energy levels, and therefore they possess high electron affinities [181]. Second, the triply degenerated C_60_ LUMO tends to reduce up to six electrons and stabilize the negative charge [182]. Third, ultrafast photo-induced electron transfer from conjugated polymers to C_60_ and its derivatives were observed by Sariciftci et al. and Yoshino and co-workers independently. They found that the electron transfer was on a time scale of ca. 50 femtoseconds, which is significantly faster than any other competing photophysical method that exists [183,184]. Finally, C_60_ derivatives also show very high electron mobilities [185].

The first bilayer heterojunction device based on conjugated polymer **MEH–PPV** and C_60_ was reported by Sariciftci et al., which delivered only a PCE of 0.04% and was slightly improved compared to pristine **MEH–PPV** single layer-based solar cells [186,187]. In order to overcome the exciton diffusion length limitation, a revolutionary development then came in the 1990s with the introduction of bulk heterojunction (BHJ) architecture.

### 5.3. Bulk Heterojunction

This architecture was invented by Yu and co-workers in 1995, where the conjugated polymer and fullerene derivatives are blended together as the active layer of an OPV device, and thus the distance that excitons migrate is dramatically reduced, and concomitantly the D–A interfacial area is significantly increased [31,188]. The photo-generated excitons are able to dissociate to free holes and electrons more efficiently compared to the previous architecture; thus the efficiency of charge separation is improved and leads to enhanced efficiency. Compared to the previous architecture where the A and D materials were consecutively placed on top of each other and could contact the cathode and anode electrodes selectively, the BHJ requires two channels for transporting electrons and holes to the electrodes. Therefore, the D and A domains should form a bicontinuous network with nano-scale morphology for efficient charge transport and collection after exciton dissociation [189]. As a result, the BHJ devices are strongly affected by the nano-morphology of the photoactive layer (Section 9) [190].

**Figure 10:**
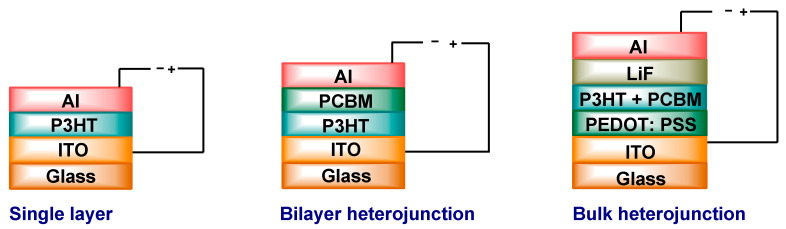
The polymer solar cell architectures.

The BHJ PSC device structure consists of several components, as illustrated in Figure 10 [191]. A transparent positive electrode, typically ITO, coated on a glass substrate is commercially available. In addition, a buffer layer of poly(3,4-ethylenedioxythiophene)-polystyrene sulfonate (**PEDOT:PSS**) is placed between the ITO electrode and the photoactive layer [192]. This layer smoothens out the ITO surface and also facilitates the hole extraction [193]. The active layer is comprised of a blend of polymer D and fullerene A and is coated on the top of the buffer layer. Finally, a negative electrode, such as Al, Mg, or Ca, which is used as a cathode, evaporates on the active layer. An electron transport layer (ETL) such as LiF is commonly inserted between the negative electrode and the photoactive layer [194].

## 6. Principle Work of Polymer Solar Cells

**Figure 11:**
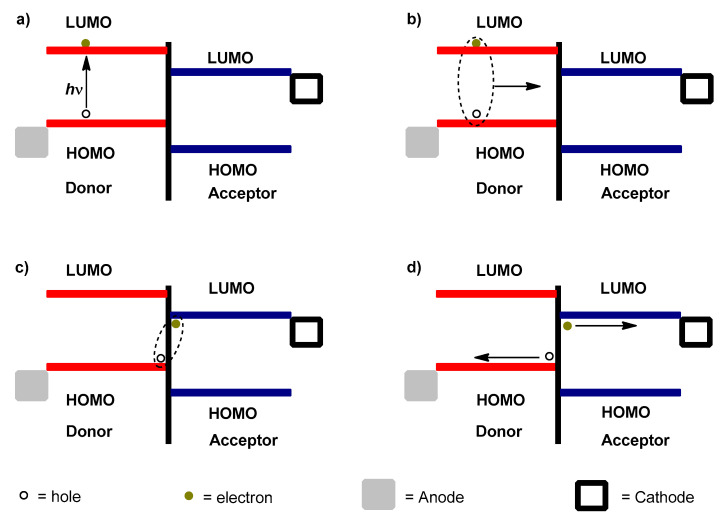
The schematic diagram for working principle of polymer solar cells.

Generally, four fundamental steps are involved in the process of charge generation from incident photons in polymer solar cells [195] (Figure 11): (a) upon the absorption of photons, electrons in the donor are promoted from the HOMO to the LUMO energy level and leave holes in the HOMO, which lead to the creation of excitons [196]; (b) the excitons subsequently diffuse to the D–A interface [169]; and (c) the electrons are transported from the LUMO energy level of the D to the LUMO energy level of the A. The electrons and holes are on acceptor and donor phases, respectively, and they are strongly joined by coulomb attractions as geminate pairs [197]. The final step (d) is the dissociation of these geminate pairs into free holes and electrons, and then the holes and electrons migrate towards the anode and cathode electrodes through donor and acceptor domains, respectively, and they are then collected (d) [198].

## 7. Characterization of Polymer Solar Cells

**Figure 12:**
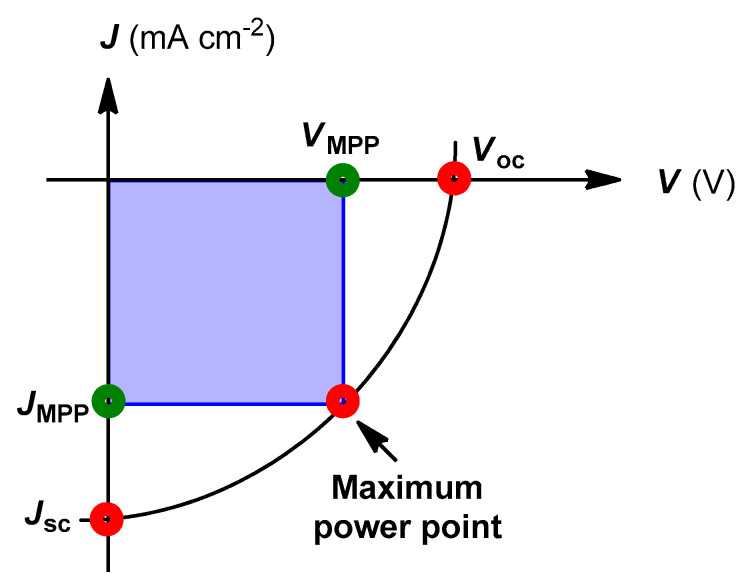
*J*–*V* curve for bulk heterojunction (BHJ) polymer photovoltaics.

Typical current–voltage (*J*–*V*) characteristics for BHJ PSCs under illumination is shown in Figure 12.

The most important performance parameters for the PSCs are short circuit current density (*J*_sc_), open circuit voltage (*V*_oc_), fill factor (*FF*), and power conversion efficiency (PCE). *J*_sc_ is the maximum current that a photovoltaic cell can generate under short circuit conditions. It is determined by the intersection of the graph with the ordinate of the *J*–*V* curve at zero bias. *V*_oc_ is the maximum voltage that a photovoltaic device can produce. It is determined by the intersection of the graph in the abscissa of the *J*–*V* curve at which current is equal to zero under open circuit conditions. *FF* is calculated by the ratio between theoretical power outputs (*V*_MPP_*J*_MPP_) at the maximum power point to the absolute power (*V*_oc_ *J*_sc_) (Equation (1)).

(1) $$FF=\;\frac{P_{Max}}{V_{oc\;}J_{sc}}=\;\frac{V_{MPP\;}J_{MPP}}{V_{oc\;}J_{sc}}$$

The estimated PCE is the ratio of *P*_out_ to the *P*_in_, where *P*_out_ and *P*_in_ are the power out and power of the incident light, respectively (Equation (2)).

(2) $$PCE=\;\frac{P_{out}}{P_{in}}=\;\frac{FF\;\;V_{oc}\;J_{sc}}{P_{in}}$$

## 8. Designing Conjugated Polymers for Photovoltaic Applications

Wudl et al. studied several soluble conjugated polymers for photovoltaic cells, for instance **PPV** derivatives (**MEH–PPV** and **MDMO–PPV)** and **rr-P3HT** [199]. The photovoltaic performance of **MEH–PPV:PC_61_BM** composite gave about 1% efficiency, which was a major step in terms of conjugated polymers [200]. In 2001, a BHJ photovoltaic cell based on an **MDMO–PPV:PC_61_BM** blend achieved a benchmark PCE of 2.5% [201]. Wienk et al. fabricated a BHJ photovoltaic cell based on **MDMO–PPV** and **PC_71_BM** that resulted in a higher PCE of 3% under AM1.5 [202]. **PPV** derivatives have deep HOMO energy levels of −5.4 eV, and as a result, the BHJ devices can provide *V*_oc_ as high as 0.82 V. Further improvement of these polymers is limited because of relatively low hole mobility and a large band gap [203] (ca. 2.0 eV), which restricts J_sc_ to 5–6 mA cm^−2^. Therefore, the highest PCE reported for this system was 3.3% [204]. Benefitting from a lower E_g_ (ca. 1.9 eV) and good hole mobilities [142,205], **P3ATs**, especially **rr-P3HT**, have become one of the most representative organic photovoltaic polymer donors. In 2002, a BHJ solar cell based on **P3HT:PC_61_BM** was fabricated and exhibited a maximum *J*_sc_ of 8.7 mA cm^−2^, which corresponded to very high external quantum efficiency (EQE) above 75% at the absorption peak. In addition, an internal quantum efficiency (IQE) close to 100% was reported for the same blend [206]. Due to extensive efforts of several groups all over the world, PCEs of around 4–5% have been reported with the **P3HT:PC_61_BM** blend [207,208,209,210].

**Figure 13:**
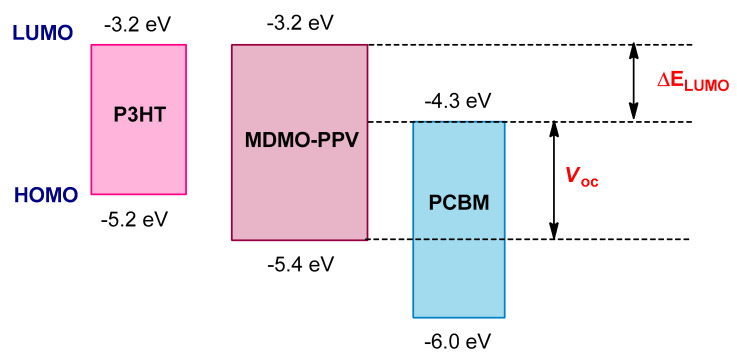
The band structures of **P3HT** and **MDMO–PPV** relative to **PCBM**.

The large E_g_ of **P3HT** (~ 1.9 eV), which can harvest only in the narrow range of the solar spectrum between 350–650 nm, is one of the main factors that limits the OPV efficiency of **P3HT**, since the peak of photon flux density from the solar terrestrial radiation is positioned at ~1.77 eV (ca. 700 nm) [211,212]. Furthermore, it has a relatively high HOMO energy level that limits the *V*_oc_ to ~0.6 V for **P3HT:PC_61_BM**-based BHJ solar cells (Figure 13) [213,214].

### 8.1. Optimization of HOMO–LUMO Energy Levels and Optical Band Gaps

In order to efficiently absorb solar energy, the absorption spectrum of polymer D should be optimally matched to the solar spectrum to maximize *J*_sc_ and also the PCE [214,215]. Therefore, it is necessary to synthesize narrow E_g_ polymers with high extinction coefficients [215]. Two strategies can be used for reducing the E_g_ of the donor polymers. Firstly, the HOMO energy level of the D can be raised, consequently resulting in decreased *V*_oc_. It worth noting that the *V*_oc_ is proportional to the energy difference between the LUMO of the A and the HOMO of the D (Figure 13) [216,217,218]. Alternatively, the LUMO level of the polymer can be lowered, and also the ΔE_LUMO_, which is the difference between LUMO levels of polymer and fullerene, which should be in the range of 0.3–0.5 eV. The value of ΔE_LUMO_ is higher than the exciton binding energy, which is important to provide efficient electron–hole pair dissociation at the polymer/fullerene interface and to promote charge carrier separation (Figure 13) [168,219,220]. However, this energy offset in the **P3HT:PC_61_BM** system is too large and results in lost energy [214].

When **PC_61_BM** is used as the acceptor (LUMO approximately −4.3 eV), the LUMO energy level of the polymer should be between −3.7 and −4.0 eV [221]. In addition, the polymer must have good air stability with a low-lying HOMO level, which would help to exhibit a higher *V*_oc_ and a higher PCE in the BHJ photovoltaic device. Therefore, the HOMO level of the polymer should be between −5.2 and −5.8 eV, and the optimal band gap should be between 1.2 and 1.9 eV [221]. Furthermore, the polymer should have a high hole mobility in order to increase *J*_sc_ and *FF* [197,222]. For the solution processability of the BHJ photovoltaic devices, the polymer should have an appropriate solubility in organic solvents, which is blended with the fullerene acceptor. Finally, the polymer should form an optimized morphology with a fullerene acceptor in the active layer with a bicontinuous network on a nanoscale to enhance the *J*_sc_ and *FF* of the polymer solar cell devices [223].

### 8.2. Strategies for Band Gap Tuning

**Figure 14:**
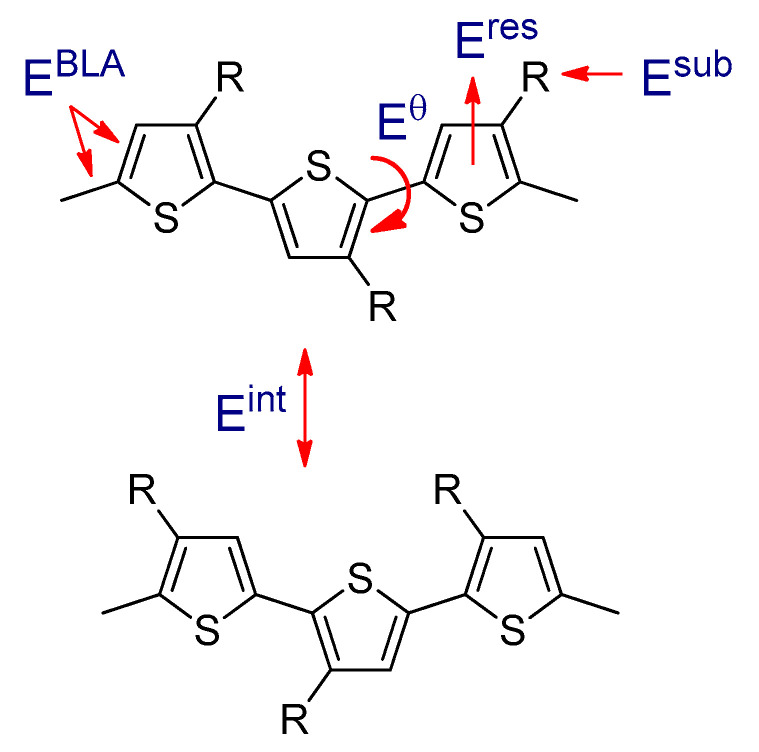
The parameters that affect the band gap of the polymer.

In order to achieve high efficiency organic photovoltaic cells, tuning the LUMO and HOMO levels of polymer and fullerene derivatives is crucial. The energy difference between the LUMO and HOMO levels of the polymer is called the band gap (E_g_), and it is influenced by several factors, including bond length alternation energy (E^BLA^), aromatic resonance energy (E^res^), torsional angle energy (E^θ^), substituents energy (E^sub^), and intermolecular interactions energy (E^int^) (Equation (3) and Figure 14) [26,224].

(3) $$E_{g}{=\;E}^{\mathrm{BLA}}{+\;E}^{\mathrm{res}}{+\;E}^{\theta }{\;+\;E}^{\mathrm{sub}}{+\;E}^{\mathrm{int}}$$

E**^BLA^** represents the major contribution to the band gap, and it is the difference between single and double bond lengths [225]. Minimizing the BLA can be achieved by increasing the quinoid form along the conjugated polymer chain, and consequently the E_g_ is reduced.

E**^res^** is associated with the aromatic resonance energy of the conjugated polymers that contain aromatic monomers, and it can be explained as the difference between the π-energy of the aromatic conjugated polymer and a structure with localized single and double bonds. Low E^res^ leads to a narrow band gap polymer [219].

E**^θ^** is related to the torsional angle (θ) between adjacent aromatic units, and one useful strategy to minimize this angle is by increasing the planarity of the conjugated backbone. Planarization can be achieved by reducing the steric hindrance between adjacent units. For example, **rr-P3ATs** adopt a more planar structure and give a high delocalization of the π-electrons, and as a result, their band gaps will be reduced. However, in **ri-P3ATs**, the alkyl side chains twist the backbone, decrease the conjugation length, and consequently increase the band gap (Figure 5) [226].

E**^sub^** is the influence on the LUMO and HOMO levels of the polymer by attaching the substituents. These levels of the polymers could be altered by introducing electron donating (ED) or electron withdrawing (EW) substituents, respectively. ED substituents, such as alkyl or alkoxy groups, elevate the HOMO level. However, EW substituents such as CN or NO_2_ lower the LUMO level. Raising the HOMO and lowering the LUMO levels leads to reduction of the band gap of the polymer [212].

Recent developments have increased the efficiency of OSCs to above 13%, an impressive achievement, through collaborative attempts in rational material design and production, particular device engineering, and essential comprehension of device physics. Through these attempts, numerous design principles for the conjugated donor polymers employed in such solar cells have emerged, containing optimized the side-chain engineering, a conjugated backbone with careful selection of building blocks, and substituents. Amongst all of the substituents, fluorine is perhaps the greatest prevalent one; enhanced device characteristics with fluorination have commonly been reported for an extensive range of conjugated polymers, especially donor–acceptor (D–A)-type polymers. Zhang et al. examined the influence of fluorination on the performance of solar cells as a function of the fluorination position (on the acceptor unit or on the donor unit), aiming to outline a perfect comprehension of the advantages of the substituent [227].

E**^int^** is determined by intermolecular interactions between the polymer backbones. In the solid phase, the chains are more ordered than in solution, and consequently the band gap is reduced [228].

**Figure 15:**
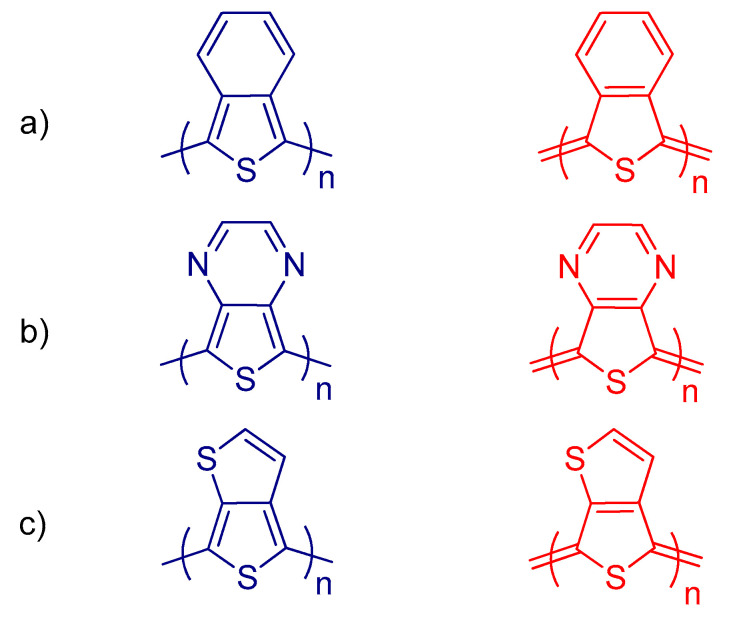
The structures of aromatic (**left**) and quinoid (**right**) forms of (**a**) **PITN**, (**b**) **PTP**, and (**c**) **PTT**.

One of the most efficient strategies to narrow the band gap is stabilizing the quinoid structure of the conjugated polymer backbone. Polythiophene (**PT**) has a large band gap (~2 eV), since it has a pronounced single bond character between the thiophene repeating units and results in large **E^BLA^** [229]. One effective method to decrease the E_g_ of **PT** is the fusion of thiophene moiety at the 3,4-positions with another aromatic unit that has higher resonance energy (E**^res^**). For example, polyisothionaphthene (**PITN**) is formed by fusion of the thiophene unit (E**^res^** = 1.26 eV) with a benzene ring (E**^res^** = 1.56 eV); the benzene ring maintains the aromaticity and the synchronous thiophene unit adopts a quinoid structure (Figure 15) [225,230]. Consequently, the E_g_ of the resulting **PITN** is lowered to 1.10 eV, which is around 1.0 eV, and it is lower than the corresponding **PT** [231]. Other low band gap polymers, such as poly(thieno[3,4-*b*]pyrazine) (**PTP**) (E_g_ = 0.95 eV) and poly(thieno[3,4-*b*]thiophene) (**PTT**) (E_g_ = 0.8–0.9 eV), were similarly synthesized by fusing thiophene with other heterocyclic rings such as pyrazine and thiophene, respectively (Figure 15) [232,233,234].

**Figure 16:**
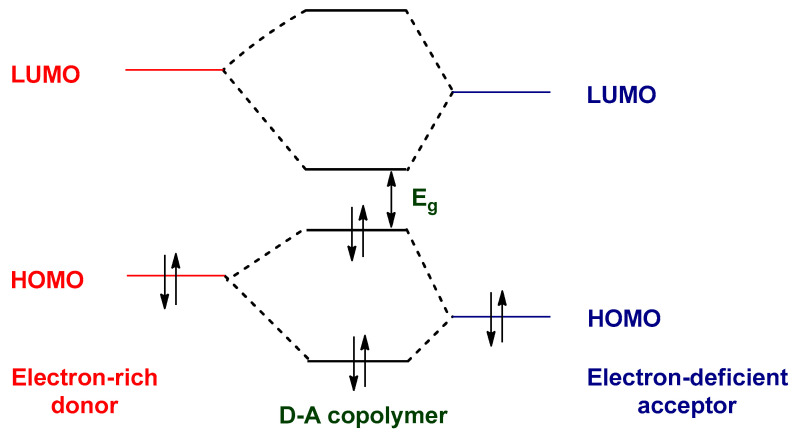
The orbital mixing between the D and the A moieties in the D–A copolymer.

The most successful approach to reducing the E_g_ of the polymers is to design donor–acceptor (D–A) copolymers, which contain D and A monomers [235,236,237]. The strong push–pull driving force facilitates electron delocalization through intramolecular charge transfer (ICT) from D monomer to A monomer [238]. Consequently, the double bond character is increased between the donor and acceptor units, reduces the E**^BLA^**, and leads to narrowing of the band gap [239]. According to molecular orbital theory, the HOMO level of the D unit hybridizes with the HOMO level of the A unit to produce two new HOMO energy levels in the D–A copolymer [240]. Similarly, the LUMO level of the D moiety mixes with the LUMO level of the A moiety to generate two new LUMO energy levels in the D–A copolymer. One of the two new HOMOs and LUMOs is higher in energy than the two initial HOMOs and LUMOs, and the other one is lower than them. Hence, the higher HOMO of the D moiety and lower LUMO of the A moiety leads to reduction of the optical band gap (Figure 16) [241].

By using this D–A approach, numerous new D–A copolymers have been developed for OPV applications with efficiencies near to or even higher than that of **P3HT** (Table 1 and Table 2).

Yang and co-workers reported the **PBDTT–DTBT** copolymer containing benzo[1,2-*b*:4,5-*b’*]dithiophene (**BDT**) as a donor unit and dithienylbenzothiadiazole (**DTBT**) as an acceptor unit (Table 1) [242]. The **PBDTT–DTBT** has an E_g_ of 1.75 eV with a deep HOMO level of −5.31 eV. The **PBDTT–DTBT:PC_71_BM**-based devices exhibited a high PCE of 5.66% [242].

Yang et al. reported a first D–A copolymer based on alternating **BDT** as a donor building block and ester substituted thieno[3,4-*b*]thiophene (**TT**) as an acceptor moiety, denoted as **PTB1** (Table 1) [243]. This copolymer was synthesized via Stille polymerization, and it has an E_g_ of 1.62 eV. The BHJ solar cells based on **PTB1:PC_61_BM** showed a PCE of 4.76%. The PCE increased to 5.6% when **PC_71_BM** was used as the acceptor [243]. Using the same polymer backbone, the effects of substituents and chain lengths were further investigated. Yu and co-workers synthesized a series of **PTBs** (Table 1). **PTB3** was synthesized by replacing the alkoxy side chains on the **BDT** moiety with alkyl chains [244]. The HOMO energy level of the resulting polymer was lowered to −5.04 eV compared to −4.9 eV for **PTB1**. As a result, the *V*_oc_ for the polymer increased to 0.72 V, and the blend of **PTB3:PC_61_BM** showed a PCE of 5.85%. By the introduction of a fluorine atom at the 3-position of **TT**, a new polymer (**PTB4**) was synthesized with an *n*-octyl side chain on the ester group and 2-ethylhexyloxy on the **BDT** moiety. The HOMO energy level of the resulting polymer was further lowered to −5.12 eV with respect to **PTB3**. Consequently, the *V*_oc_ was slightly enhanced to 0.74 V and the BHJ devices fabricated from **PTB4:PC_61_BM** exhibited a higher PCE of 6.1% [244].

Yu et al. further studied **PTB7** using 2-ethylhexyl side chains on both **BDT** and **TT** moieties [245]. BHJ PSCs fabricated from **PTB7:PC_71_BM** offered a very impressive PCE of 7.4% [245,246]. A similar polymer in the same series (**PBDTTT–CF**) was reported by Li and co-workers in which the ester group on the **TT** unit was replaced by a ketone group (Table 1) [247]. The higher PCE of 7.7% was obtained in devices based on **PBDTTT–CF:PC_71_BM** [248].

Following these outstanding results for the **PBDTTT** copolymers, the **BDT** unit copolymerized with another electron accepting unit, thieno[3,4-*c*]pyrrole-4,6-dione (**TPD**), to form **PBDTTPD 3-**copolymers (Table 2). The first **PBDTTPD** copolymer was reported by Leclerc et al. [249]. They used a straight alkyl chain on the **TPD** unit and branched alkoxy chains on the **BDT** unit to enhance the solubility of the polymer, which was synthesized through Stille polymerization. The resulting polymer exhibited an E_g_ of 1.80 eV, which was higher than that of **PBDTTT** copolymers. In addition, the **PBDTTPD** copolymers had lower HOMO energy levels than **PBDTTT** copolymers, and consequently higher *V*_oc_ values could be expected. **PBDTTPD** blended with **PC_71_BM** showed a PCE of 5.5% [249]. Soon after, Jen [250] and Frechet [251] groups synthesized the same polymer but with higher *M*_n_ values of 33,000 and 35,000 g mol^−1^, respectively, than the previously reported polymer, which has an *M*_n_ value of 13,000 g mol^−1^. The polymer was fabricated with **PC_71_BM** in the Jen group and exhibited a PCE of 4.1% [250]. The Frechet group enhanced the PCE to 6.8% when they blended the polymer with **PC_61_BM** [251]. Xie et al. reported two **PBDTTPD** copolymers with two different alkoxy chains on **BDT** units and the same alkyl chain on the **TPD** unit. **PBDTTPD1** and **PBDTTPD2** were synthesized with high *M*_n_ values of 43,500 and 91,100 g mol^−1^, respectively, and they had the same E_g_ of 1.84 eV (Table 2) [252]. BHJ solar cells of **PBDTTPD1** and **PBDTTPD2** with **PC_71_BM** gave a PCE of 3.42 and 4.79%, respectively, with *V*_oc_ values as high as 0.90 V [252]. Later on, a series of **PBDTTPD** copolymers was reported by Beaujuge et al., and it was found that attaching 2-ethylhexyloxy chains on the **BDT** unit and an *n*-heptyl chain on **TPD** improved the performance of the polymer [253]. A remarkable PCE of 8.5% was achieved for the **PBDTTPD**:**PC_71_BM** blend (Table 2) [253].

## 9. Morphology

Even though the electron donor polymers have suitable optical and electronic properties, the polymers must be blended with a fullerene-based electron acceptor such as PCBM to form the active layer in the BHJ structure. The overall efficiency of BHJ photovoltaic cells depends on the nanomorphology of the photoactive layer.

The degree of phase separation between the polymer and fullerene A is crucial, and ideal BHJ morphology has the domain sizes within the exciton diffusion length, which is in the orders of tens of nanometers [254]. If the domain sizes between the two components are too small, the transport of the free charge carriers will be strongly hindered due to the poorly conductive pathways for charge collection. As a result, free electrons and holes recombine before reaching their respective electrodes via bimolecular recombination. This type of morphology is unfavorable, and the PCE of the device could be low due to inefficient charge collection, which causes loss of *J*_sc_ and *FF*. In the case of large domains, few excitons are able to reach the polymer:fullerene interface and subsequently separate to free charge carriers. The performance of this type of morphology is limited due to insufficient exciton dissociation, which causes loss of *J*_sc_ and *FF*. Therefore, to achieve the optimal nanomorphology of the photoactive layer, controlling the degree of phase separation and feature size is essential during the fabrication of BHJ solar cells.

The nanomorphology of the photoactive layer can be affected by several processing parameters such as the choice of solvent(s) for spin casting film, thermal and solvent annealing, solvent additive, and blend composition [255].

### 9.1. Choice of Solvent(s)

The first important point is the choice of solvent(s) on the nanomorphology of polymer:PCBM film and the performance of the device. Shaheen et al. showed the impact of solvent on the morphology of the photoactive layer and device efficiency of the **MDMO–PPV:PC_61_BM** blend film [201]. A PCE of 0.9% was achieved when toluene (TO) was used for casting the film, whereas the efficiency was dramatically increased to 2.5% when TO was replaced by chlorobenzene (CB). The size of the fullerene domains within the resulting films changed enormously with the choice of solvent [256]. For instance, Yang and co-workers observed that the size of PCBM clusters was less than 100 nm in CB-cast films, while in TO-cast films PCBM-rich domains were micrometer-sized [257]. The solubility of PCBM in CB was higher than in TO.

Liu and co-workers fabricated photovoltaic cells from blends of **MEH–PPV:C_60_** with different solvents (CB, *o*-dichlorobenzene (DCB), xylene (XY), tetrahydrofuran (THF), and chloroform (CF)) [258]. It was claimed that the CB, DCB, and XY solvents induced better contacts between the polymer and C_60_ molecules, resulting in larger *J*_sc_ and lower *V*_oc_ than by using THF and CF solvents. Rispens et al. fabricated solar cells from **MDMO–PPV:PC_61_BM** blends by changing the solvents from XY through CB to DCB, and they compared the surface topology of the active layers [259]. They found that the phase separation was decreased from XY through CB to DCB. The PCE of 3% was obtained for a device made from CB with a significant improvement of *J*_sc_ and *FF*. The PCE of 6.1% was reported for **PCDTBT** and **PC_71_BM** photovoltaic cells prepared from DCB, which was higher than those devices processed from CF or CB [25]. This is probably due to DCB providing optimal phase separation relative to CF and CB solvents.

**Scheme 26:**
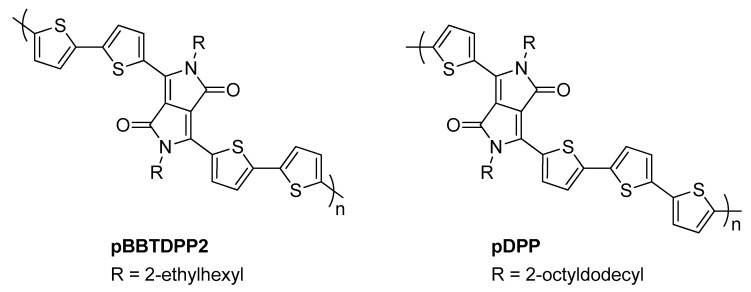
Structures of **pBBTDPP2** and **pDPP**.

Some remarkable results have been reported using mixed solvents. For example, a study on a blend of **PFDTBT** with **PC_61_BM** fabricated by incorporating a small amount of CB in CF (CF:CB = 80:1, *v*/*v*) displayed a significantly enhanced *J*_sc_ compared to devices prepared from neat CF. However, a reduction in *J*_sc_ was exhibited when devices made from CF were mixed with XY or TO [260]. Janssen and co-workers showed the effect of mixed solvents on the nanomorphology of the active layers and the efficiencies of the photovoltaic devices containing low band gap polymer, **pBBTDPP2** blended with **PC_61_BM** (Scheme 26) [261]. The device processed from CF:DCB (4:1, *v*/*v*) provided the highest PCE of 3.2% compared to devices made from neat CF (1.1%) or DCB (2.9%). This was due to the large difference in boiling points and vapor pressures of the solvents; the evaporation rate of DCB is slow and allows the polymer to crystallize. In addition, the devices prepared from DCB alone and DCB:CF show small features less than 100 nm using atomic force microscopy (AFM) measurements of the blends, while devices processed from CF alone display large domains of several hundreds of nm. Similarly, Liu et al. used CF alone and DCB:CF (1:16 and 1:4, *v*/*v*) for fabricating low band gap polymer, **pDPP** mixed with **PC_71_BM** [262]. A low PCE of 1.05% was achieved when CF was used alone. The performance of the devices was significantly enhanced by gradually increasing the DCB content. The PCE was increased to 4.16% in the case of DCB:CF (1:16, *v*/*v*) and the highest PCE of 5.62% was obtained in DCB:CF (1:4, *v*/*v*). The higher performance is the result of higher *J*_sc_ due to improved crystallinity and morphology of **pDPP:PC_71_BM** blends casting from mixed solvents.

### 9.2. Thermal Annealing

**Scheme 27:**
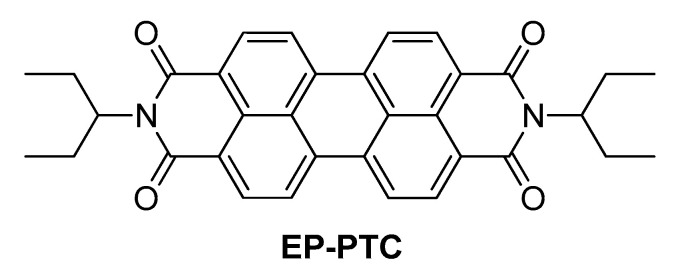
Structure of **EP**–**PTC**.

Thermal annealing has proven to play an important role to control the nanomorphology of certain types of active layer materials. Dittmer and co-workers observed the effects of thermal treatment on **P3HT** blended with organic small molecule dye, *N*,*N*′-bis(1-ethylpropyl)perylene-3,4:9,10-tetracarboxylic diimide **(EP–PTC)** (Scheme 27) [263]. The EQE was improved after annealing at 80 °C for one hour compared to the non-annealed device. This improvement was related to enhancement of the crystallinity of **P3HT** upon thermal annealing, which resulted in increased carrier mobility in the blend.

Camaioni and co-workers reported that the efficiency of **P3HT:fulleropyrrolidine** solar cells could be increased three- to four-fold under mild thermal treatment at 55 °C for 30 min [264]. Padinger et al. reported a postproduction treatment (after deposition of the cathode electrode) of **P3HT:PC_61_BM** devices, and the PCE of the device was improved to 3.5% [265]. Since then, extensive efforts have been devoted to optimize thermal annealing via carefully controlling temperature and time in order to improve the morphology of the photoactive layer and increase the efficiency of **P3HT:PC_61_BM** devices; PCEs around 5% were reported [208,210]. Chirvase et al. studied the influence of thermal treatment on the nano-morphology and PCE of **P3HT:PC_61_BM** devices [266]. They concluded that the absorption of **P3HT** was red-shifted in the blend films upon annealing the devices. Mihailetchi and co-workers also reported similar phenomena [267].

The structural and optical properties of the **P3HT:PC_61_BM** composite were investigated using grazing-incidence X-ray diffraction (GIXRD) measurements upon thermal annealing [268]. The **P3HT** backbone orientation became parallel to the substrate; however, their side chains were oriented perpendicularly to the substrate after thermal annealing. In addition, upon thermal annealing, fullerene molecules were redistributed and diffused into larger domains. Consequently, the absorption of **P3HT** was shifted to longer wavelengths due to an increase in crystallinity. A detailed investigation using transmission electron microscopy (TEM) and electron diffraction confirmed that upon thermal annealing of **P3HT:PC_61_BM** blend films, the length of fibrillar crystals of the **P3HT** phase was increased [269]. As a consequence, a large interfacial area was formed, and the efficiency of charge generation was enhanced due to the improvement of charge transport for the blends upon annealing and yielded higher photovoltaic device efficiencies. Moreover, **P3HT:PC_61_BM** blends of several other systems such as **PCPDTBT:PC_61_BM** and **PDTSBT:PC_71_BM** showed higher efficiencies after thermal annealing [270,271].

### 9.3. Solvent Annealing

Solvent annealing (or slow growth) is another effective technique to alter the nano-morphology of the active layers. This is done by placing cast films in contact with solvents or their vapors in a partially closed container such as a covered glass Petri dish, which slows the evaporation rate of the solvent [272,273,274]. Solvent annealing of **P3HT:PC_61_BM** blends produced a high degree of ordering of **P3HT** chains, and the crystallinity of the **P3HT** was improved, and the polymer chains became self-organized [207]. As a result, the absorption of **P3HT** was shifted to a lower energy region and controlled phase separation [207]. Mihailetchi and co-workers reported that the hole mobility of **P3HT** was improved 33-fold in a **P3HT:PC_61_BM** blend upon solvent annealing [275]. Li et al. investigated the rate of solvent evaporation and thermal treatment on the device efficiency of **P3HT:PC_61_BM** blends [207]. It was found that a slow evaporation rate over a 20 min period of the DCB solvent during film formation gave a PCE of 3.5%. Reduction of the evaporation time to 3 min led to a decrease in PCE to 2.80%. However, during fast solvent removal via heating the blend at 50 and 70 °C, the PCE was further dropped to 2.10 and 1.36%, respectively. In addition to solvent annealing, the device annealed at 110 °C for 20 min, and the PCE increased to 4.37%. The improved PCE was due to a high *FF* of 67.4%, self-organization of the **P3HT** chains, increased hole mobility, and better balanced electron and hole transport. The effects of solvent annealing and thermal annealing on the polymer nanoscale crystallinity, absorption, and device performance of **P3HT:PC_71_BM** blends have been studied by Chu et al. [276]. Controlling the solvent-removal rate increased the molecular ordering of the **P3HT** in the blended films, as confirmed by GIXRD. A PCE of 3.80% was achieved for **P3HT:PC_71_BM** after thermal annealing at 110 °C for slow grown film. The high efficiency was attributed to improved *J*_sc_ and *FF.* The enhancement of *J*_sc_ and *FF* was due to greater absorption and higher charge carrier mobility, respectively. Shrotriya et al. investigated the impact of self-organization by controlling solvent-removal rate on the performance of **P3HT:PC_61_BM** blends [277]. It was found that the *J*_sc_ and *FF* were improved for slow growth processes of the active layer. This enhancement was attributed to increased exciton generation and dissociation efficiency, enhanced carrier mobility, and highly balanced charge transport.

### 9.4. Solvent Additives

The utilization of solvent additives is an alternative method for solvent and thermal annealing for controlling the nanomorphology of the photoactive layer in organic photovoltaic cells. Studies of the addition of additives to host solvent during film processing of the blend were first reported by the Bazan group [278]. It was demonstrated that the photoconductivity, carrier mobility, and lifetime in **P3HT:PC_61_BM** BHJ films were significantly increased by adding octanethiol (5% by volume) to the TO host solvent, and the structural order was enhanced [278]. Peet et al. reported that after the addition of a few volume percentages of 1,8-octanedithiol **(ODT)** (2.5% by volume) into the **PCPDTBT:PC_71_BM** system, the photovoltaic device efficiency was almost doubled from 2.8 to 5.5% [279]. Soon after, Lee et al. showed in a systematic study the addition of 1,8-di(X)octanes (X = CN, SH, I, Br, Cl, and CO_2_CH_3_) to CB host solvent for fabricating **PCPDTBT:PC_71_BM** BHJ solar cells [280]. It was found that 1,8-diiodooctane **(DIO)** was the best additive, since the PCE of the solar cells was increased from 3.4 to 5.1%. It was also demonstrated that alkanedithiols selectively dissolved the fullerene in the CB host solvent [280], since alkanedithiols have higher boiling points than CB, allowing the fullerene molecules to stay longer in the solution than the polymer during film processing [280]. Consequently, the morphology of thin films can be manipulated by selection of various additives and their concentrations to control the phase separation between donor and acceptor molecules [280].

Upon addition of the **ODT** to the **P3HT:PC_61_BM** blend, the crystallinity of the **P3HT** chains was increased [281]. In addition, higher hole mobility was achieved by adding octanethiol to the **P3HT:PC_61_BM** composite [278]. However, no increase in hole mobility was observed when field effect transistor (FET) measurements were used for the **PCPDTBT:PC_71_BM** active layer by incorporating **ODT** [279]. Moreover, X-ray diffraction (XRD) data indicated that the **PCPDTBT:PC_71_BM** films were amorphous with or without **ODT** processing [279]. Therefore, the increased device performance must result from the improved interpenetrating network, better percolation pathways for holes and electrons, and also the electron mobility was significantly improved in the films when processed with **ODT** [282].

### 9.5. Blend Composition

The blend composition of the polymer and fullerene compounds is an important parameter that influences the morphology and device performance of the system. In the case of **PPV** derivatives with **PCBM**, the best device performance was reported by taking the weight ratio of polymer:PCBM (1:4) to provide a suitable nanoscale phase separation between the two components, an efficient charge transport, and a reduced recombination [201,283,284]. In contrast to the PPV-based devices, **P3HT:PCBM** composites require lower fullerene content for optimum photovoltaic cell efficiencies. Chirvase et al. studied the influence of the **P3HT:PCBM** weight ratio on the nanomorphology and the solar cell performance of **P3HT:PCBM** blends [266]. It was found that the *J*_sc_ and EQE of the blends significantly increased with decreases of the PCBM loading, and the maximum values were attained at 50 wt% of **PCBM**. The highest PCE was achieved for device based on **P3HT:PCBM** blends (1:1, *w*/*w*).

Ma et al. recorded a PCE of about 5% using a **P3HT:PCBM** (1:0.8, *w*/*w*) active layer [210]. Shrotriya et al. studied the impact of **P3HT:PCBM** weight ratios on the absorption spectra of films and the photovoltaic performance of **P3HT:PCBM** BHJ solar cells [285]. It was confirmed that the absorption spectra of the films were blue-shifted at about 63 and 80 nm when the amount of **PCBM** was 67 and 75 wt%, respectively. The maximum absorption wavelength for the blend was achieved with 50 wt% **PCBM**. It was also found that the *J*_sc_ values were increased using a lower amount of PCBM, and the maximum value of 9.9 mA cm^−2^ was attained with **P3HT:PCBM** (1:1, *w*/*w*). An efficiency of 3.85% was obtained due to an increase of the absorption in the low energy regions and better charge carrier transport in the **P3HT:PCBM** (1:1) composite.

## 10. Novel Acceptor Materials

As mentioned earlier, ΔE_LUMO_ can be lowered by upshifting the LUMO energy level of the fullerene in order to maximize the *V*_oc_ of **P3HT**-based PSCs as well as to increase the PCE of the device (Figure 13). Koster et al. calculated the ultimate PCE of a **P3HT:PC_61_BM** BHJ solar cell versus ΔE_LUMO_ and band gap [220]. First, the effect of lowering the ΔE_LUMO_ to 0.5 eV by raising the LUMO level of the acceptor (i.e., the band gap of the **P3HT** unchanged) was predicted to achieve an efficiency of 8.6%. This efficiency increase resulted from an increase of *V*_oc_ value. Second, it was shown that lowering the E_g_ of the **P3HT** to 1.5 eV and lowering its LUMO level (i.e., *V*_oc_ value unchanged) led to an efficiency of 6.6%. This efficiency increase resulted from the enhancement of the *J*_sc_ value. Finally, Koster and co-workers calculated the energy conversion efficiency of a **P3HT:PC_61_BM** BHJ solar cell to 10.8% for optimized ΔE_LUMO_, band gap, layer thickness, and high hole mobility [220].

**Figure 17:**
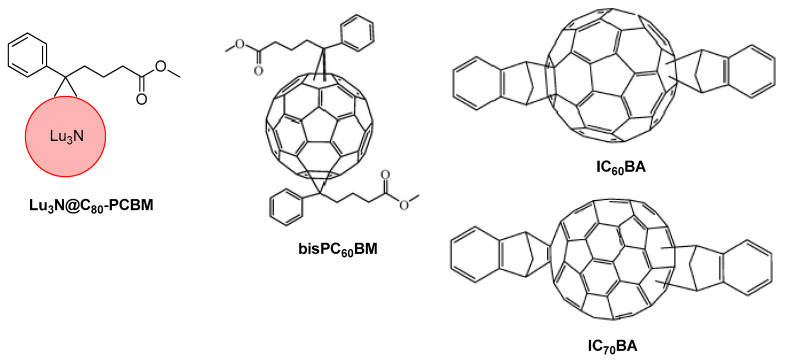
Structures of novel fullerene derivatives.

Several novel fullerene derivatives were developed and utilized as acceptors and tested in BHJ PSCs, such as lutetium-based endohedral fullerenes (**Lu_3_N@C_80_**), PC_60_BM-bisadduct (**bisPC_60_BM**), indene-C_60_bisadduct (**IC_60_BA**), and indene-C_70_bisadduct (**IC_70_BA**) (Figure 17) [286,287,288,289].

In 2009, Ross et al. reported an endohedral C_80_-fullerene derivative (**Lu_3_N@C_80_–PCBM**) [286]. A PCE of **P3HT:Lu_3_N@C_80_–PCBM** BHJ solar cells reached 4.2%, which was higher than that of the **P3HT:PC_61_BM** system (3.4%). Both devices had the same *J*_sc_ and *FF*, but the former blend had a higher *V*_oc_ relative to the latter cell benefitting from a higher LUMO level of **Lu_3_N@C_80_–PCBM** than **PC_61_BM**. A bis-adduct analogue of PCBM (**bisPC_60_BM**) had a higher LUMO energy level of about 0.1 eV compared to that of **PC_61_BM**. A **P3HT:bisPC_60_BM**-based solar cell exhibited a higher PCE of 4.5%, which was higher than that of a **P3HT:PC_61_BM**-based solar cell (PCE of 3.8%) [287]. Li et al. reported a new bis-adduct fullerene derivative (**IC_60_BA**) with stronger absorbance in the visible region compared to **PC_61_BM**, and the LUMO level of **IC_60_BA** upshifted to −3.74 eV, which was higher than **PC_61_BM** [288]. The photovoltaic devices including **P3HT:IC_60_BA** showed an excellent PCE of 5.44% compared to **P3HT:PC_61_BM**-based polymer solar cells (PCE of 3.88%), under similar conditions. After device optimi**z**ation by the same group, the PCE of 6.5% was achieved for BHJ photovoltaic cells containing **P3HT:IC_60_BA** [290]. Li and co-workers further designed and synthesized new bis-adduct fullerene (**IC_70_BA**), which displayed stronger light absorption in the visible region than **PC_61_BM** [289]. The LUMO level of **IC_70_BA** was higher than that of **PC_61_BM** and **PC_71_BM**. Consequently, the *V*_oc_ and the PCE of the photovoltaic devices including**P3HT:IC_70_BA** significantly improved (0.84 V, 5.64%) compared to **P3HT:PC_61_BM** (0.59 V, 3.55%) and **P3HT:PC_71_BM** (0.58 V, 3.96%), respectively. Sun et al. further optimized the devices based on **P3HT:IC_70_BA** BHJ solar cells, and the PCE reached 6.69% [291]. More importantly, Guo and co-workers further optimized the photovoltaic devices based on a **P3HT:IC_70_BA** blend, which showed a remarkable PCE of 7.40% [292].

## 11. Summary and Perspectives

Polymer solar cells (PSCs) have received considerable attention as a renewable energy source due to their benefits such as lightweight, flexible devices, and solution processing. The PCE of BHJ PSCs, where the photoactive layer involves a blend of donor and acceptor, has dramatically increased in the last few years. To achieve high PCEs within these devices, the conjugated polymer should have a deep HOMO level to increase *V*_oc_, a low energy band gap in order to efficiently absorb the solar energy resulting in higher *J*_sc_, a high absorption coefficient, and high hole mobilities [293]. The most efficient strategy to construct low band gap polymers relies on the use of alternating D and A moieties along the backbone of conjugated polymers. The charge transfer between D and A can effectively control the band gap of D–A copolymers. Introducing electron donating substituents, such as alkyl or alkoxy groups, can elevate the HOMO level of donor monomer. Moreover, electron withdrawing substituents such as CN, F, and NO_2_ can lower the LUMO level. Raising the HOMO and lowering the LUMO levels leads to reducing the band gap of the polymer.

Using this strategy, several types of D–A copolymers have shown excellent PCEs. The challenge for organic solar cells is associated with the preparation of a material with high absorption for the solar spectrum and a simple processing method. The fraction of sunlight that can be absorbed is specific for each material and varies with its chemical structure. Band gap and molecular energy level controls are crucial for device performance. The mismatch of the polymer absorption spectra and the solar irradiance spectrum is one of the reasons for low efficiencies of devices. Chemical modification of the semiconducting polymers structure is a common approach that leads to tuning of the band gaps. The extension of conjugation degree leads to an enhancement, in terms of intensity, and red-shift of the absorption spectra of conjugated polymers, as the collection of the radiation is broadened and more photons can be absorbed, thereby contributing to the energy conversion.

Even though the D and A have ideal optical and electronic properties, the overall efficiency of BHJ photovoltaic cells depends on the nanomorphology of the photoactive layer. The nanomorphology of the photoactive layer can be affected by several processing parameters such as the choice of solvent(s) for spin casting film, thermal and solvent annealing, solvent additive, and blend composition.

## Figures and Tables

**Table 1:** Structures and photovoltaic performance of **PBDTT–DTBT**, **PTBs**, and **PBDTTT–CF** copolymers in BHJ PSCs.

Polymer	*V*_oc_ (V)	*J*_sc_ (mA cm^−2^)	FF (%)	PCE (%)	Ref.
**PBDTT–DTBT** 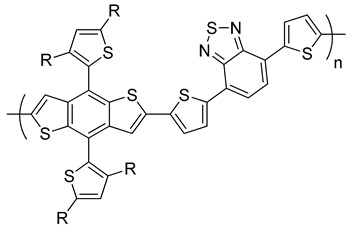 R = octyl	0.92	10.70	57.5	5.66	[242]
**PTB1** 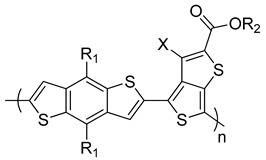 X = H, R_1_ = octyloxy, R_2_ = dodecyl	0.58	12.50	65.4	4.76	[243]
**PTB3:** X = H, R_1_ = octyl, R_2_ = 2-ethylhexyl	0.72	13.90	58.5	5.85	[244]
**PTB4:** X = F, R_1_ = 2-ethylhexyloxy, R_2_ = octyl	0.74	13.00	61.4	6.1	[244]
**PTB7:** X = F, R_1_, R_2_ = 2-ethylhexyl	0.74	14.50	68.9	7.4	[243,246]
**PBDTTT–CF** 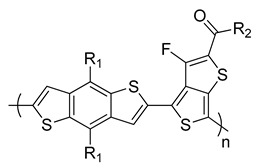 R_1_ = 2-ethylhexyloxy, R_2_ = heptyl	0.76	15.20	66.9	7.7	[247]

**Table 2:** Structures and photovoltaic performance of **PBDTTPD** copolymers in BHJ PSCs.

Polymer	*V*_oc_ (V)	*J*_sc_ (mA cm^−2^)	FF (%)	PCE (%)	Ref.
**PBDTTPD** 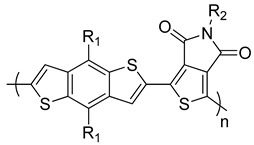 R_1_ = 2-ethylhexyloxy, R_2_ = octyl	0.85 0.84 0.85	9.81 9.80 11.50	66.0 49.5 68.0	5.5 ^a^ 4.1 ^b^ 6.8 ^c^	[249] [250] [251]
**PBDTTPD1** R_1_ = dodecyloxy, R_2_ = 2-octyldodecyl	0.93	6.58	56.0	3.42 ^d^	[252]
**PBDTTPD2** R_1_ = 2-ethylhexyloxy, R_2_ = 2-octyldodecyl	0.91	10.34	51.0	4.79 ^d^	[252]
**PBDTTPD** R_1_ = 2-ethylhexyloxy, R_2_ = heptyl	0.97	12.60	70.0	8.5 ^e^	[253]

^a^ Leclerc et al., ^b^ Jen group, ^c^ Frechet group, ^d^ Xie et al., ^e^ Beaujuge et al.
